# Supplementary material for: Perceptions on artificial intelligence-based decision-making for coexisting multiple long-term health conditions: protocol for a qualitative study with patients and healthcare professionals
Source: BMJ Open. 2024 Feb 1;14(2):e077156. doi: 10.1136/bmjopen-2023-077156 (PMC10836375; doi:10.1136/bmjopen-2023-077156)
Supplement: Supplementary data [file bmjopen-2023-077156supp001.pdf]

Supplementary Material 1

List of 63 conditions considered when determining eligible patients with 2 or more conditions.

Heart failure
Atrial fibrillation (AF)
Stroke
Hypertension
Ishaemic heart disease/ coronary artery disease (IHD)
Peripheral vascular disease (PVD or PAD)
Heart valve disorders
Aortic aneurysm
Type 1 diabetes
Type 2 diabetes
Chronic kidney disease (CKD)
Depression
Anxiety
Bipolar disorder
Eating disorder
Schizophrenia
Post traumatic stress disorder (PTSD)
Autism
Drug/alcohol misuse
Alcoholic liver disease
Non-alcoholic fatty liver disease (NAFLD)
Other (or non-specified) chronic liver disease
Inflammatory bowel disease (IBD)
Irritable bowel syndrome (IBS)
Dementia
Parkinson's disease
Epilepsy
Cancer (excluding BCC)
Haematological cancer (leukaemia/lymphoma/ myeloma)
Asthma
Chronic obstructive pulmonary disease (COPD)
Obstructive sleep apnoea (OSA)

Bronchiectasis and cystic fibrosis
Eczema
Allergic rhinitis (hayfever)
HIV/AIDS
Osteoporosis
Osteoarthritis
Rheumatoid arthritis
Gout
Systemic lupus erythematosus (SLE)
Sjogren’s disease
Systemic sclerosis
Polymyalgia rheumatica / Giant cell arteritis (PMR/GCA)
Fibromyalgia/ chronic fatigue (CFS)
Polycystic ovarian syndrome (PCOS)
Endometriosis
Hypothyroidism
Hyperthyroidism
Addison’s disease
Multiple sclerosis
Vision impairment long term
Hearing impairment long term
Meniere’s disease
Peripheral neuropathy
Intellectual disabilites
Down syndrome
Pernicious anaemia
Sickle cell anaemia
Psoriasis
Psoriatic arthritis
Interstitial lung disease
Haemochromatosis

Supplementary Material 2

Information sheet/ Invitation letter

Practice address/contact details

<<Patient Address>>

<<Date>>

OPTIMIsing therapies, disease trajectories, and AI assisted clinical management for patients Living with complex multimorbidity (OPTIMAL study)

Your Unique Reference Code is: CPRD *<<Global ID>>*

Dear *<<Patient name>>*

We would like to invite you take part in a research study. The research team are hoping to speak to people who have multiple (four or more) long-term health conditions to discuss their thoughts about how artificial intelligence (AI) may help them and their health care professionals manage these conditions. They will also be speaking to health care professionals to help understand their views.

Before you decide whether you would like to take part in an interview, please read the enclosed participant information sheet that explains the study in more detail and what would happen if you agree to take part.

If you would like to participate in an interview, please contact a member of the research team whose details are given below.

The research team:
OPTIMAL Team
Institute of Allied Health Research, University of Birmingham, B15 2TT

For more information, contact:
[name, email and contact number of study lead]

Thank you for taking the time to consider taking part in the study.

Yours sincerely,

<<GP sign off>>

UNIVERSITY OF BIRMINGHAM

NHS
National Institute for Health Research

OPTIMIsing therapies, disease trajectories, and AI assisted clinical management for patients Living with complex multimorbidity (OPTIMAL)

Participant information sheet: patient interviews

Introduction

We would like to invite you to take part in a research study. Before you decide to take part, we would like to explain why we are doing this study and what your involvement will mean for you. Please take time to read this information sheet and discuss it with others if you wish. Ask us if there is anything that is not clear or if you would like more information before deciding whether or not to take part. Our contact details are at the end of this information sheet.

What is the study about?

The study aims to use artificial intelligence (AI) to produce computer programmes and tools that will help improve the treatment and choice of medications in patients with clusters of multiple long-term conditions (we say these people have complex multimorbidity or cMM). Artificial intelligence (or AI) is a computer system that can conduct tasks that would normally need human intelligence. There are examples of AI being used in our everyday lives, with applications and software such as Spotify, Amazon and BBC iplayer. These ‘apps’ and on-line websites function by predicting what kind of music, TV programmes, or general purchases we may like or want. They do this by using a computer programme that can observe what categories and kinds of TV, Music etc. we watched or bought in the past and use this data to predict what we are a likely to choose in the future.

The machine learning branch of AI is also used in things like self-driving cars, language translations and it is being developed for use in healthcare. Newer AI methods makes it easier to process large amount of health data in a short time. These AI methods can give doctors and patients information that may help improve the care of people with multiple long-term health conditions

AI can be used in healthcare to help guide the diagnosis of long-term health conditions, plan the best treatment strategies, and predict the next health condition that people might develop. This is especially relevant for people who have several different health conditions

because the guidelines that doctors and healthcare professionals may use for one condition does not routinely consider other health conditions.

AI-based decision-making tools (computer programs) for managing multiple health conditions may help patients and healthcare professionals make more well-informed shared decisions. Patients and healthcare professionals may see many benefits to the use of AI in healthcare. However, they may be concerned this could be harmful or that it could affect the relationships between healthcare professionals and patients. We want to speak to patients and healthcare professionals to understand their views about AI in healthcare. It is important in healthcare research that we reflect these experiences in any future plans for the use of AI in a healthcare setting.
Data collected from this study will give new insights to how healthcare professionals and people with multiple long-term health conditions view AI-based decision-making tools. It will also tell us which factors in the computer program are important to them and what options they prefer. This information will also be used to help to develop how these tools are used in clinical practice.

How will we do this?

1. By using artificial intelligence (AI) methods with electronic health records to generate data models that tell us how the different mixes of conditions arise over time and how certain drugs can make this better or worse.

2. By asking people with cMM and doctors about their knowledge and views about using AI to make decisions about health care.

3. By using AI computer techniques to combine data, and together with the input from people with cMM and doctors, develop a computer program. This will predict which drug we should give and when we should give it to someone with cMM to reduce risk and bring about maximum benefit. It will also tell us what disease people may get next. These predictions will be based upon data gathered from a large number of patients who have similar conditions and prescribed medication.

4. By examining the best way to present information in the AI tool to people with cMM and doctors by asking them about what is important to them and what options they prefer.

Our team includes patients, public members, and world leading experts from universities with expertise in biology, AI, medicine, health service research, public health, and general practice. Working as a multidisciplinary team we hope to improve the health and care of people with cMM.

Why have I been asked?

You have been invited because we would like to talk to you about your experiences living with four or more long-term health conditions.

Do I have to take part?

Taking part in this study is completely optional, the decision about whether to take part is up to you. Whether you decide to take part or not will have no effect upon your healthcare and treatment and you can leave the study at any time, without giving a reason.

What will happen to me if I take part?

If you choose to take part, you can contact the research team by telephone or email (details below). The researcher can provide more information about the study, and answer any questions you may have.
Please note that because we would like to interview a range of people depending upon certain characteristics, we will ask you for some basic details including your age, ethnicity, gender and number of health conditions). It may take a number of weeks before we can to arrange an interview date. In the event that we do not require your assistance, we will contact you to let you know.
The interviews will take place either face-to-face or remotely using videoconferencing or telephone at a time convenient to you. The interview should last for around 60 minutes, although you can ask for a break or stop the interview at any time.

The interviewer will ask questions about your thoughts on how AI informed decision making (the use of computer programmes) compares with doctors making decisions about your health.

We will also show you an example of a patient with several long-term conditions and how their health care has been managed using a computer programme, for example, how the computer programme has helped decide what medications they may need. We will ask you what kinds of things are important to know if a computer is informing these kinds of decisions about the medications you are prescribed your health in general.

If you agree to take part, we would like to audio record the interview using a secure recording device. After the interview, the recording will be written down (in a transcript) but all names and place names will be removed and no comments will be linked to you or any other person. The recording and transcript will be kept completely confidential using a study ID code and only the University evaluation team will have access to them.

At the end of the interview, you will be asked if you would be willing to be contacted at a later date to consider involvement in the second stage of the study. This stage will aim to explore preferences in terms of how computer programmes for AI directed clinical decision making are presented. For example, how the computer displays information, and what things patient think are important to see.

What are the possible benefits of taking part?

Although there is no direct benefit to you if you take part in an interview, the interviews will give us important information that should help patients with cMM along with the doctors who manage their conditions.

Will I receive any financial reimbursement for taking part?

You will be offered a £15 voucher (Amazon or Love 2 Shop) as a thank you for taking part in the interview.

What are the possible risks and disadvantages of taking part?

It may be upsetting to talk about living with multiple long-term conditions and your health care experiences. If this happens, we can stop or pause the interview at any time. We have put together a list of organisations that you can get in contact with who may be able to help. Many people have said that they find it helpful to talk to researchers about their experiences of living with conditions.
Taking part in the interview will take some of your time. However, you can choose a time and date that is suitable for you.
In light of COVID-19, we will adhere to current government guidelines and local procedures in order to minimise the risk of exposure. Support for participants will be provided to set up an online conferencing link, or by telephone according to participant preference. Researchers will have evidence of vaccination and/or negative results from a lateral flow test prior to interview if face-to-face.

What if there is a problem?

If you have any concerns, please speak to a member of the research team in the first instance, you can get in touch with us using the contact details at the end of this information sheet. We will do our best to answer your questions. If your concerns are not addressed and you wish to make a formal complaint, you can refer to the Patient Liaison Services (PALS)
0121 424 0808 PALS@uhb.nhs.uk.

Will my taking part in the study be kept confidential?

All information collected about you for this study will be subject to the General Data Protection Regulation and Data Protection Act 2018 for health and social care research and will be kept strictly confidential.

All audio-recordings will be kept for 10 years after the end of the study and then destroyed. Documentation and data from this part of the study will be securely stored at the University of Birmingham for 10 years.

How will we use information about you?

The University of Birmingham is the Sponsor for this study and this means that the University of Birmingham are responsible for looking after your information and using it properly. University of Birmingham and the NHS will keep identifiable information about you for at least 10 years after the study has finished, to allow the results of the study to be verified if needed. Information collected from you will include:

- Name
- Age

- Ethnicity
- Gender
- Number of long-term conditions

People who do not need to know who you are will not be able to see your name or contact details. Your data will have a unique study number instead and all information will be kept safe and secure. In the research team, you will be identified using your unique study number.

All information collected by the Sponsor, including a copy of your signed consent form, will be securely stored at the research study office at the University of Birmingham on paper and electronically and will only be accessible by authorised personnel. The only people in the University of Birmingham who will have access to information that identifies you will be people who manage the study or audit the data collection process.

The audio recordings from the interview will be transcribed by a transcription company which has been approved for transcription of medical data. If you agree to take part in the interview study, your name will not be on the recording and we will remove your name from the interview transcripts to keep your identity confidential. Direct quotes may be used in publications but these will be numbered and anything that could identify you will be removed. Nothing that you say will be fed back to the doctors and nurses involved in your care as coming from you.

What are your choices about how your information is used?

You can choose to stop taking part in the study at any time, without giving a reason, but we will keep information about you that we already have. If you agree to take part in this study, you will have the option to take part in future research using your data saved from this study. To safeguard your rights, we will use the minimum personally identifiable information possible. You can find out more about how your information will be used at https://www.birmingham.ac.uk/privacy/index.aspx.

The NHS and the University of Birmingham will use your name and contact details to contact you about the research study, and make sure that relevant information about the study is recorded and to oversee the quality of the study. Individuals from the University of Birmingham and regulatory organisations may look at your research records to check the accuracy of the research study.

All individuals who have access to your information have a duty of confidentiality to you. Under the provisions of the General Data Protection Regulation (GDPR) 2018, you have the right to know what information the Trial Office has recorded about you. If you wish to view this information, or find more about how we use this information, please contact the University of Birmingham's Data Protection Officer at the address below.

Where can you find out more about how your information is used?

If you would like more information on your rights, would like to exercise any right or have any queries relating to our processing of your personal data, or if you wish to make a complaint about how your data is being or has been processed, please contact:

The Data Protection Officer, Legal Services, The University of Birmingham, Edgbaston, Birmingham B15 2TT
Email: dataprotection@contacts.bham.ac.uk
Telephone: +44 (0)121 414 3916

You can also find out more from www.hra.nhs.uk/information-about-patients/ and by reading the information available here **www.hra.nhs.uk/patientdataandresearch.**

What will happen if I don't want to carry on with the study?

Your participation is completely voluntary. If you choose to take part but change your mind later, you are free to leave the study at any time, without giving a reason, and without your healthcare being affected. If you wish to withdraw from the study, please contact a member of the team (details are at the end of this document), However, please note that if you decide to withdraw more than 2 weeks after participating in an interview, any data already collected may still be used in the study.

What will happen to the results of the research study?

The information we collect will be analysed and the results will be presented in several ways:

- A short written summary of the results will be available on the OPTIMAL website hosted by the University of Birmingham, or can be sent out to participants should they prefer.
- A detailed report will be written and will be available for participants on the OPTIMAL website hosted by the University of Birmingham, or can be sent out to participants should they prefer.
- We will publish the results in academic journals.

Your details will not be shared at any time and you will not be identified in any of the results from the research.

Who is organising and funding the research?

The research study is funded by the National Institute for Health Research (NIHR). The research is sponsored and insured by the University of Birmingham.

Who has reviewed the study?

All research in the NHS is looked at by an independent group of people called a Research Ethics Committee (REC), to protect your interests. This study has been reviewed and given favourable opinion by South Central – Hampshire B Research Ethics Committee (REC

Reference: 22/SC/0210). Patients and public Involvement (PPI) members have been involved throughout all stages of the research study.

What happens next?

If you would like to participate in an interview, please contact a member of the research team whose details are given below.

The research team:
OPTIMAL Team
Institute of Allied Health Research,
University of Birmingham
B15 2TT

For more information, contact:
[name, email and contact number for study lead]

Supplementary Material 3

Study Title: **OPTIMIsing therapies, disease trajectories, and AI assisted clinical management for patients Living with complex multimorbidity (OPTIMAL study)**

Dear Colleague

We are writing to you on behalf of a team of researchers based at the University of Birmingham who are undertaking the above named study which aims to use artificial intelligence (AI) to produce computer programmes and tools that will help improve the treatment and choice of drugs in patients with clusters of multiple long-term conditions (complex multimorbidity or cMM).

As part of this study, the team are hoping to speak to healthcare professionals who care for patients with multiple (or more than four) long-term conditions (complex multimorbidity) to discuss their thoughts about how artificial intelligence (AI) may help professionals and patients manage these conditions. We will also be speaking to patients with long-term conditions to get their views.

We have attached a copy of the participant information sheet (PIS), to provide more details about the study. Before you decide whether you would like to take part in an interview, please read the enclosed participant information sheet that explains the study in more detail and what would happen if you agree to take part. If this is something you may be interested in taking part in, please email the address below.

Yours sincerely,

[name, email and contact number of study lead]

Institute of Applied Health Research
University of Birmingham
B15 2TT

Supplementary material 4

Case study for participants with multiple health conditions

Harinderjeet is 53 years old and owns a plumbing business with several employees. Harinderjeet has asthma, chronic lung disease, migraine, osteoarthritis (joint pain) and angina (chest pain). His work is very important to him as he feels responsible for his employees and wants to develop the business further. Most notably, his work helps to keep him distracted from this health conditions and pain.

He was diagnosed with asthma when he was 42 years, angina and osteoarthritis at age 45 years and then with lung disease two years later. As a result of his diagnoses he gave up smoking and has been smoke-free for the past five years. His GP told him he should exercise more and he tries to but the pain he experiences in his joints makes it difficult and he hardly has the time with running his business. When he last saw his GP last week he had his weight measured and his BMI was 29 kg/m^2 and his doctor said that he should think about losing some weight. Harinderjeet thinks that the worsening of some of his symptoms and health conditions are caused by side effects of some of the drugs he takes; he takes approximately 20 tablets a day.

Harinderjeet spends a large amount of time going to doctor and hospital appointments, both planned and unplanned contacts with the health care system about once a week. His is mainly in contact with his GP who will regularly review the drugs for his pain and asthma but then has separate appointments at the hospital for his lung disease and arthritis. He also sees a clinical nurse specialist for his angina. He started developing some severe pain in his tummy and told the doctor at the arthritis clinic who told him that it wasn't related to his arthritis and he should talk to his GP about it.

Q1. How does Harinderjeet's experience of being diagnosed with multiple health conditions compare with your experience? Explain some of the similarities and the differences.

Q2. How does Harinderjeet's experience of feeling that some of the worsening of his symptoms and being diagnosed with other health conditions are due to the medicines he is taking compare with your experience? Explain some of the similarities and the differences.

Case vignette for healthcare professionals - Janet

Janet is a white English woman aged 72 years who is a retired secretary living at home on her own.

From her records you can see:

She had an NSTEMI 3 years ago.

She has hypertension diagnosed over 10 years ago, type 2 diabetes diagnosed 6 years ago, and she has chronic kidney disease stage 3a.

BMI - 32.2 kg/m2.
Average home blood pressure is 134/82.
Renal function is stable with an eGFR of 55.
Diabetes is stable and reasonably well controlled with a Hba1c of 52.

She is a non-smoker and drinks occasionally. She does minimal exercise.

Her medications are:

Ramipril 10mg - ischaemic heart disease, CKD, diabetes, hypertension

Amlodipine -5mg hypertension

Glyceryl trinitrate spray - as required. - ischaemic heart disease.

Aspirin 75mg - ischaemic heart disease

Bisoprolol 10mg - ischaemic heart disease, hypertension

Atorvastatin 80 mg - ischaemic heart disease

Metformin - modified release 500mg BD - diabetes.

In today's consultation:

Her husband died 10 years ago, and she has a supportive family who live nearby but struggles with loneliness, especially at night time. She had bereavement counselling at the time of his death which helped her a lot. She used to go to church regularly until about 6 months ago and

has been a member of a choir for many years. However, over the past 6 months she has lost interest in going to these things any more (or joining the online versions even though she knows how). She has very poor sleep, reduced appetite, she can't concentrate on a book or TV programme. She feels quite "empty" and tired a lot of the time. She often thinks it might be better if she did not wake up the next day, but she does not have any plans for suicide. **You have diagnosed her with moderate to severe depression and she is now asking whether she could try some medication.** She knows her son has has been taking fluoxetine for many years for his depression and he found this really helpful. She is also willing to try some CBT.

Q1: What factors in the history (demographics, symptoms, past medical history, medications) would you consider when deciding how to manage her depression? (pt's age, sleep/appetite problems, other health problems, other medications, patient preference)

Q2: What factors or resources would you normally use to help make complex decisions like this? (own experience, NICE guidance, other guidance, patient preference?)

Q3: Assuming you decide to start an antidepressant, what would you recommend for Janet, and why?

Q4: The computer algorithm says Janet should take sertraline with a PPI, do you agree? (this is just an example, and I don't know if that would be the answer and whether it will give an explanation or not)

Q5: How would you feel about using the computer algorithm to make a decision like this compared with your usual practice? If you agree with the algorithm/ if you don't agree?

Very comfortable Mostly comfortable Not sure Mostly uncomfortable Very uncomfortable

Why?

Relevant NICE guidelines copied below:

From NICE guidelines; https://cks.nice.org.uk/topics/depression/management/new-or-initial-management/

Which antidepressants are recommended for people with chronic physical health problems?

- Prescribe a generic selective serotonin reuptake inhibitor (SSRI), such as sertraline or citalopram unless these are contraindicated or there is a significant drug interaction.
    - Do not use citalopram or escitalopram in people who are taking medication that could prolong the QT interval.
- For a brief summary on which antidepressants may be prescribed for people with chronic physical health problems, see Table 1. For more detailed information see appendix 16 of the full NICE guideline.

Table 1. Antidepressants recommended for people with a chronic physical health problem.

Medication being taken for a chronic physical health problem	Antidepressants that should not normally be offered	Suitable options
Nonsteroidal anti-inflammatory drugs (NSAIDs)	Selective serotonin reuptake inhibitor (SSRI) or a serotonin noradrenaline reuptake inhibitor (SNRI) (if no suitable alternative can be found, offer gastroprotection)	Mirtazapine, moclobemide, reboxetine, or trazodone
Aspirin	SSRIs and SNRIs (if no suitable alternative can be found, offer gastroprotection)	Mirtazapine.<br>When aspirin is used alone, consider trazadone or reboxetine.

Supplementary material 5

OPTIMIsing therapies, disease trajectories, and AI assisted clinical management for patients Living with complex multimorbidity (OPTIMAL study)

Interview guide: Patient Interview

This is the starting topic guide. The overarching objectives will remain the same, but questions and prompts will be developed as interviews are undertaken to incorporate any important themes emerging from the interviews.
Before the interview begins

- Ensure the participant has read the information leaflet
- Ensure the participant has had the opportunity to ask any questions about the research including issues about confidentiality, the findings of the research and where the research will be disseminated before being asked to agree to each item on the consent form.
- Start audio-recording
- Go through each item on the consent form and record their verbal consent. Explain that you will send/email a copy of the consent form for their records. They should already have a copy of the participant information sheet with details about the study, how to withdraw etc
- Explain that they don't have to answer all the questions just because they have consented to the interview, and that they can take a break or stop the interview at any time.
- Explain that you are there to understand more about their experiences and that they will have some time at the end of the interview to talk about any other issues that are important to them that may not have been covered by the questions.
- Check that they are happy to continue to be audio-recorded.
- Begin the interview.

TOPICS TO BE COVERED IN THE INTERVIEW

Patient's reflections on living with/managing long-term conditions

Could you tell me about your health conditions? Prompts – how long have you had conditions, how have they developed over time

What are your experiences of managing patients your long-term conditions? Prompts – medication, self-management, navigating primary/specialist care, relationships with clinicians/HCPs

Have you experienced any difficulties managing your conditions? Prompts -

- Side-effects from medication (impact on appetite, understanding which medications are for which condition; remembering to take medication)
- Multiple appointments for different conditions; opportunities to see GP/specialists; information sharing;

- Impact of conditions upon other activities e.g. work, time to socialise, leisure activities, access
- Impact upon relationships

Acceptability of AI in clinical decision making

Attitudes and understanding about AI in general terms (briefing guide of discussion points about AI)

Have you any thoughts about AI and how it is used in everyday activities?
Do you have any experience of AI being used to manage your healthcare?

Attitudes towards and perspectives about AI health care in managing long term conditions

In this study interview we want to explore the advantages and limitations of AI directed clinical decision making and compare this with doctors making decisions.

How do you feel about how clinical decisions are made about your healthcare? E.g. do you feel involved in decisions?

Do you think the use of AI (computer programs) in healthcare decision making could be beneficial? If so, how could it be used? If not, what are the reasons why?

Do you think that AI (computer programs) could improve clinical decision making?

Do you think that AI (computer programs) could help you manage your health conditions? If so, how may this benefit you and what would 'good' look like?

In which situations do you think AI (computer programs) should not be used?

What would your concerns be, if any?

How do you feel that using AI (computer programs) for clinical decision making may compare to clinician/patient making decisions about your health?

Can you think of ways AI (computer programs) making decisions about your care may be preferable to clinicians making decisions about your care?

Can you think of ways clinician/patient guided decisions about your care are preferable to AI directed decisions about your care? E.g. continuity of relationship; importance of relationship with HCP

What kind of questions might you want to ask before agreeing to treatment that was directed by AI?

What kind of things may influence your choice between AI or clinician/patient guided decisions about your care?

How confident would you feel if prescription decisions were directed by AI (computer program/predictive algorithm) rather than your doctor?
Very comfortable Mostly comfortable Not sure Mostly uncomfortable Very uncomfortable

Why?

Stage two: Vignettes – validity of AI in clinical decision making

We are developing a tool that will detect what other conditions a patient may develop in the future based upon their current health conditions. It will also provide information about what sort of medications should be prescribed (to achieve maximum health benefits and reduce side-effects etc)

Participants will have been sent a copy of the case vignette prior to the interview

We will present to the participant, a simulated patients disease clusters, to compare how aspects of the patient care management fit in with their own experiences:
The case vignette will present a simulated patients disease clusters, with 4 health conditions showing:

- How conditions developed
- What other conditions the patient developed
- Impacts of conditions upon their lifestyle and relationships etc
- How medications were managed
- How overall health management is navigated via the AI algorithm

How does the patient's experience of being diagnosed with multiple health conditions compare with your experience? Explain some of the similarities and the differences.

How does the patient's experience of feeling that some of the worsening of their symptoms and being diagnosed with other health conditions are due to the medicines he is taking compare with your experience? Explain some of the similarities and the differences.

What other things would you want to know about this patients to inform decisions about management of their treatment e.g lifestyle factors?

Would it be useful to know for example what conditions may develop in the future?

Would this impact upon things like lifestyle changes? E.g. dietary/exercise/self-monitoring regime

How would you feel about a computer programme predicting the medications you may benefit most from rather than your doctor?

Very comfortable Mostly comfortable Not sure Mostly uncomfortable Very uncomfortable

Does this case simulation reflect your own kind of experiences and preferences in terms of how your cMM are managed?

What do you think are the most important things that researchers need to consider when developing computer programmes to manage patient care?

Conclusion of interview

Now we've talked a bit about how AI could be used, how do you feel about it?

Thinking back to the difficulties you talked about in managing treatments, do you think AI could have an effect on this?

Thank you. That was my last question.

Is there anything you would like to add about the things we talked about but have not covered in the interview?

Any questions from interviewee

Reminder of study contact details and signposting

OPTIMIsing therapies, disease trajectories, and AI assisted clinical management for patients Living with complex multimorbidity (OPTIMAL):

Interview guide: Staff Interview

This is the starting topic guide. The overarching objectives will remain the same, but questions and prompts will be developed as interviews are undertaken to incorporate any important themes emerging from the interviews.

Before the interview begins

- Ensure the participant has read the information leaflet
- Ensure the participant has had the opportunity to ask any questions about the research including issues about confidentiality, the findings of the research and where the research will be disseminated before being asked to agree to each item on the consent form.
- Start audio-recording
- Go through each item on the consent form and record their verbal consent. Explain that you will send/email a copy of the consent form for their records. They should already have a copy of the participant information sheet with details about the study, how to withdraw etc
- Explain that they don't have to answer all the questions just because they have consented to the interview, and that they can take a break or stop the interview at any time.
- Explain that you are there to understand more about their experiences and that they will have some time at the end of the interview to talk about any other issues that are important to them that may not have been covered by the questions.
- Check that they are happy to continue to be audio-recorded.
- Begin the interview.

TOPICS TO BE COVERED IN THE INTERVIEW

<u>Managing patients with four or more long-term conditions</u>?

What are your experiences of managing patients with four or more long-term conditions? Prompt – what factors are important to consider when compared with patients with single diseases , comorbidties, other meds

What difficulties do patients with four or more long-term conditions have in managing their conditions and making decisions about treatment options?

Acceptability of AI in clinical decision making

Attitudes and understanding about AI in general terms (briefing guide of discussion points about AI) such as Amazon/ BBC iPlayer/ Spotify giving recommendations based on the existing data they have collected?

Do you have any experience of using AI in clinical practice?

Attitudes towards and perspectives about AI health care in managing cMM.

How do you think using AI directed clinical decision making may compare to usual practice?

Thinking about the impact on health care professionals ***and*** patients:

Thinking specifically about managing physical health conditions: Prompt? Diabetes, COPD, heart failure, CKD:

What are your thoughts on advantages and disadvantages of AI directed clinical decision making compared with your usual practice?

How do you think patients feel about AI directed clinical decision making?

What do you think patients may see as the advantages/ disadvantages of AI directed clinical decision making?

Are there examples where either using AI or using usual practice might be preferable?

What kind of things may influence your choice?

What are your thoughts on using AI directed clinical decision making with patients who have mental health conditions, e.g. depression/ anxiety?

What are your thoughts about using AI directed clinical decision making to make decisions about prescribing in physical health conditions (e.g., choosing the best medication for diabetes).

What are your thoughts about using AI directed clinical decision making to make decisions about prescribing treating mental health conditions (e.g., choosing the best antidepressant).

How confident would you feel if prescription decisions were directed by a predictive algorithm compared to your usual practice?

Very comfortable Mostly comfortable Not sure Mostly uncomfortable Very uncomfortable

Why?

Stage two: Vignettes – validity of AI in clinical decision making

We will present to the participant, a range of simulated patients from a range of disease clusters, to compare how aspects of the AI patient care management fit in with their own experiences a clinician:

What resources would you normally use to help make complex decisions like this? (NICE guidelines, own experience, other guidelines)

What medication changes would you recommend and why? (pt's age, sleep/appetite problems, other health problems, other medications, patient preference)

The computer algorithm says the patient should take X with the following explanation**, do you agree?**

How would you feel about using the computer algorithm to make a decision like this compared with your usual practice?

Very comfortable Mostly comfortable Not sure Mostly uncomfortable Very uncomfortable

Why?

How would you feel if the algorithm recommended a treatment you were not expecting? Would you over-ride if you disagree?

What would you do if a patient didn't want to follow the recommended treatment (i.e., would you look to over-rule it or stick by it?

Close of interview

Thank you. That was my last question. Is there anything you would like to add about the things we talked about but have not covered in the interview?

Supplementary materials 6

<u>Artificial Intelligence (AI) Background for participants</u>

Artificial Intelligence or AI is the term used to describe a computer system or algorithm that can conduct tasks that would normally require human intelligence.

There are examples of AI being used in our everyday lives, with applications and software such as Spotify, Amazon and BBC iPlayer. These 'apps' and on-line websites function by predicting what kind of music, TV programmes, or general purchases we may like or want. They do this by using a computer programme that can observe what categories and kinds of TV, music etc. we watched or bought in the past and use this data to predict what we are likely to choose in the future.

AI is also used in things like self-driving cars and language translations, and it is also being increasingly developed for use in healthcare. For example, AI has been developed that can screen the retina scans of patients with suspected diabetic eye disease. This is the leading cause of blindness in adults, and in many parts of the world, there are not enough doctors or health care professionals to undertake the work involved in diagnosing the condition. The AI trained system can diagnose the condition with the same accuracy as a trained healthcare professional.

In healthcare AI is also being developed to help us to predict how patients' health may progress in the future. It can also help to decide what are the best treatments and medications for the increasing number of people who are living with several long-term health conditions

Using the large amounts of anonymised patients' data available from GP and hospital electronic health records, AI can help us predict the life trajectories of people with multiple long-term conditions. For example, we know that people with diabetes may already have or are more likely to develop high blood pressure, heart disease and eye disease. These patients are usually prescribed medications to help manage all these conditions, but it can be difficult to ensure the best medications are prescribed for each individual patient based on the complexities of their own specific medical history, other characteristics and the variability in decisions made by different health professionals providing care.

Given the variability of factors that can influence health, having an efficient, accurate and easy to use AI programme that can take into account this variability and help 'predict' the best combination of medications, or what conditions may develop in the future may benefit patients and health care professionals.
