# Supplementary material for: Perceptions on artificial intelligence-based decision-making for coexisting multiple long-term health conditions: protocol for a qualitative study with patients and healthcare professionals
Source: BMJ Open. 2024 Feb 1;14(2):e077156. doi: 10.1136/bmjopen-2023-077156 (PMC10836375; doi:10.1136/bmjopen-2023-077156)
Supplement: Author's manuscript [file bmjopen-2023-077156.draft_revisions.pdf]

BMJ Open is committed to open peer review. As part of this commitment we make the peer review history of every article we publish publicly available.

When an article is published we post the peer reviewers' comments and the authors' responses online. We also post the versions of the paper that were used during peer review. These are the versions that the peer review comments apply to.

The versions of the paper that follow are the versions that were submitted during the peer review process. They are not the versions of record or the final published versions. They should not be cited or distributed as the published version of this manuscript.

BMJ Open is an open access journal and the full, final, typeset and author-corrected version of record of the manuscript is available on our site with no access controls, subscription charges or pay-per-view fees (http://bmjopen.bmj.com).

If you have any questions on BMJ Open's open peer review process please email info.bmjopen@bmj.com

Patients' and healthcare professionals' perceptions on artificial intelligence-based decision making for coexisting multiple long-term health conditions: A qualitative study protocol

Journal:	*BMJ Open*
Manuscript ID	bmjopen-2023-077156
Article Type:	Protocol
Date Submitted by the Author:	27-Jun-2023

Complete List of Authors:	Gunathilaka, Niluka; Ministry of Health Sri Lanka,<br>Gooden, Tiffany; University of Birmingham College of Medical and Dental Sciences,<br>Cooper, Jennifer; University of Birmingham, Institute of Applied Health Research<br>Flanagan, Sarah; University of Birmingham, Institute of Applied Health Research<br>Marshall, Tom; University of Birmingham, Public Health and Epidemiology<br>Haroon, Shamil; University of Birmingham,<br>D'Elia, Alexander ; University of Birmingham College of Medical and Dental Sciences<br>Crowe, Francesca; University of Birmingham, Institute of Applied Health Research<br>Jackson , T; University of Birmingham,<br>Nirantharakumar, Krishnarajah; University of Birmingham, Institute of Applied Health Research<br>Greenfield, Sheila; University of Birmingham, Institute of Applied Health Research

Keywords:	QUALITATIVE RESEARCH, Primary Health Care, PUBLIC HEALTH

SCHOLARONE™ Manuscripts

Patients' and healthcare professionals' perceptions on artificial intelligence-based decision making for coexisting multiple long-term health conditions: A qualitative study protocol

Niluka J Gunathilaka[1*], Tiffany E Gooden[1*], Jennifer Cooper[1], Sarah Flanagan[1], Tom Marshall[1], Shamil Haroon[1], Alexander D'Elia[1], Francesca Crowe[1], Thomas A Jackson[2,3], Krishnarajah Nirantharakumar[1], Sheila Greenfield[1]

1. Institute of Applied Health Research, University of Birmingham, Birmingham, United Kingdom
2. Institute of Inflammation and Ageing, University of Birmingham, Birmingham, United Kingdom
3. Department of Healthcare for Older People, Queen Elizabeth Hospital, Birmingham, United Kingdom

* Joint first authors

Corresponding author:

Francesca Crowe: f.crowe@bham.ac.uk, Institute of Applied Health Research, University of Birmingham, Birmingham UK

Word count: 2942

Keywords: multimorbidity; patient perspectives, clinical support, artificial intelligence, qualitative methods, shared decision-making

For peer review only

ABSTRACT

Introduction

Coexisting multiple health conditions is common among older people, a population that is increasing globally. The potential for polypharmacy, adverse events, drug interactions and causing additional health conditions complicates prescribing decisions for these patients. Artificial Intelligence (AI)-generated decision-making tools may help guide clinical decisions in the context of multiple health conditions, by determining which of multiple medication options is best. This study aims to explore the perceptions of healthcare professionals (HCPs) and patients on the use of AI in the management of multiple health conditions.

Methods and analysis

A qualitative study will be conducted using semi-structured interviews. Adults (≥18 years) with multiple health conditions living in the West Midlands of England and HCPs with experience in caring for patients with multiple health conditions will be eligible and purposively sampled. Patients will be identified from Clinical Practice Research Datalink (CPRD) Aurum; CPRD will contact general practitioners who will in turn, send a letter to patients inviting them to take part. Eligible HCPs will be recruited through British healthcare professional bodies and known contacts. Up to 30 patients and 30 HCPs will be recruited, until data saturation is achieved. Interviews will be in-person or virtual, audio recorded and transcribed verbatim. The topic guide was designed to explore participants' attitudes towards AI-informed clinical decision-making to augment clinician-directed decision-making, the perceived advantages and disadvantages of both methods and attitudes toward risk management. Case vignettes comprising a common decision pathway for patients with multiple health conditions will be presented during each interview to invite participants' opinions on how their experiences compare. Data will be analysed thematically using the Framework method.

Ethics and dissemination

This study has been approved by the National Health Service Research Ethics Committee (Reference: 22/SC/0210). The findings from this study will be disseminated through peer-reviewed publications, conferences and lay summaries.

For peer review only

STRENGTHS AND LIMITATIONS

- This study will use a UK population-based database comprising more than 30 million primary healthcare electronic records to facilitate a selection of a diverse sample of patients with multiple long-term conditions within the West Midlands in terms of key demographics and mix of co-occurring conditions.
- Including perspectives from both patients and HCPs will allow us to capture doubts, barriers or issues in using AI-based clinical support tools from the people that will be affected the most by the development and implementation of an effective AI tool.
- Use of a systematic methodology and extensive involvement of our patient and public advisory group have ensured our data collection tools are fit for purpose.
- Despite the strength of using a UK population-based database, the database may also provide limitations due to possibilities of under-representation of certain groups due to structural inequalities.
- Non-probability sampling and including patients only from the West Midlands may limit generalisability.

For peer review only

INTRODUCTION

One quarter of adults in England have two or more long-term health conditions.(1, 2) Having four or more long-term health conditions is strongly correlated with increasing age and has a significant contribution toward health service utilisation.(3) People with multiple long-term conditions are often prescribed multiple different medications (polypharmacy), which can lead to multiple side effects in addition to symptoms from their health conditions.(4) Evidence also suggests that polypharmacy can result in lower quality of life (5) and a three to four-fold increased risk in mortality among people aged 65 years or above.(6)

From the healthcare perspective, the more conditions a person has and more medications they take, the harder it is for healthcare professionals (HCPs) to consider all the factors when determining the best treatment plan, as clinical guidelines for one condition do not usually consider other existing health conditions.(7) Evidence is lacking on how to best treat health conditions for people with multiple long-term health conditions which in many cases, leads to excess treatment burden.(8) Due to limited resources for people with multiple long-term health conditions and the complex interaction between different disease conditions, primary care HCPs have identified the need for developing and adopting guidelines on how to best deliver and manage care for those with multiple health conditions.(9) Evidence based guidelines are typically established from findings of clinical trials for individual diseases; however, such trials often exclude people with multiple health conditions.(7) As a result, HCPs are provided with guidelines for individual conditions that are often too complex and heterogenous to combine or integrate to determine the best treatment option for individuals with multiple health conditions. Observational studies could be conducted using large scale population-based data (such as routinely collected electronic health records) to better understand the impact of medication on multiple health conditions; however, such studies are limited by the inability to account for individual patient medical histories, demographics (e.g. age, gender, ethnicity) and the variability in decisions made by HCPs providing care.

Artificial intelligence (AI) tools may be able to overcome some of these limitations in a way that humans cannot and may help patients and HCPs make more well-informed shared decisions. In this context, AI is a term used to describe a prediction algorithm to identify the next disease(s) and next best treatment for patients. However, for AI decision-making tools to be successfully

For peer review only

implemented and effectively used in clinical practice, it is important that HCPs and patients trust, understand and see the value of using the tool. To date, nine qualitative studies have captured patients' views on AI use in general healthcare, two of which focused on AI-based decision-making tools.(10) Results from these two studies showed that patients generally had a positive perception of AI use in healthcare, for example to support screening for diabetic retinopathy (11) and skin cancer.(12) However, there were often reservations, especially about the ability of AI to appreciate individual context and human experience. There were mixed opinions about the impact that AI may have on the clinician-patient interaction. There is limited evidence that describes patient perceptions of using AI-based tools for managing existing conditions or multiple health conditions.(13) Conversely, results from a 2021 UK-based study that included HCPs indicated mixed views around general use of AI in healthcare; specifically, within the NHS where HCPs have expressed concerns over regulatory processes, the transferability/generalisability of the tools and the lack of knowledge and experience of using AI tools.(9) Application of these technologies in the healthcare setting is still in its infancy and misconceptions surrounding this area are common.(14) Therefore, it has been difficult to harness informed insights from patients and clinicians about the use of AI in decision-making. A developed, real-world example of an AI-based tool is needed to explore informed perceptions of this technology with both patients and HCPs, and specifically regarding the management of multiple health conditions.

The OPTIMising therapies, disease trajectories, and artificial intelligence (AI) assisted clinical management for patients Living with multiple long-term health conditions (OPTIMAL) study aims to produce an AI-based tool to be used in primary care settings for planning the best treatment strategies and predicting the next health condition that people with multiple health conditions might develop. Using population-based anonymised data available from primary and secondary healthcare records, the OPTIMAL study will determine the trajectories of disease accumulations for people with multiple health conditions and the contribution of medicines on the trajectories. The team will then develop a predictive model for the next likely disease and the best treatment option in the context of multiple conditions and multiple treatment options and incorporate this into an AI-based decision-making tool that can be used by HCPs and patients for joint decision making for the best treatment plan. Input from patients with multiple health conditions and HCPs on using the AI-based tool is critical in its development and implementation to ensure any specific

For peer review only

barriers for the use of the tool can be appropriately addressed. Therefore, another component of the OPTIMAL study is a qualitative study which this protocol describes.

Theoretical framework

Theoretical frameworks can be used at all stages in qualitative research to allow for a deeper understanding of how people and the cultures and organisations they are part of operate, interact and behave.(15) Several theoretical frameworks have been used to conceptualise attitudes and barriers to the introduction of AI in healthcare settings.(9, 16, 17) Rogers' Diffusion of Innovations (DOI) theory (9) describes six elements that influence the diffusion of an innovation within a pre-existing system including: relative advantage (perception of improvements compared to the current system), compatibility with the current system, complexity of the innovation, trialability (potential for testing), potential for adaptation of the tool for unforeseen purposes and tangibility of observed effects. This has been used to model how NHS managers and researchers perceive that the characteristics of both AI technologies and the current model and culture of UK health service delivery would influence the likely introduction of AI in NHS settings. However, to explore the perspectives of general practitioners (GPs) on AI in primary care in Germany, Buck et al.(16) built on Rosenberg and Hovland's (18) previous work on the construct of attitudes to change (Figure 1). This model distinguishes the affective (emotional reactions, empathy, and feelings), cognitive (ideas and knowledge), and behavioural (extent to which attitudes predict actions and intentions to act) dimensions to attitudes to use of AI-enabled systems in healthcare.(14, 18) Individual characteristics such as age and prior experience with AI, and environmental influences such as the positive attitude from primary care professional bodies also influenced attitudes to AI. Given that the application of AI in general practice is largely hypothetical at present, current attitudes to AI from potential users are critical to the development of technologies that could then be implemented in practice. Therefore, Buck et al.'s theoretical lens will be used at the analysis stage as a framework to explore the data from HCP interviews to provide a broader conceptual understanding of how the affective, cognitive and behavioural components of attitude determine how HCPs evaluate their views on AI in managing multiple health conditions.

Through semi-structured interviews (SSIs), this qualitative study aims to understand patients' and HCPs' perceptions of the advantages and disadvantages presented by AI-informed decision-

For peer review only

making compared to clinician directed decision-making for the management of multiple health conditions.

METHODS AND ANALYSIS

Study design and study participants

This qualitative study will be conducted using SSIs (19) with two key groups of stakeholders. The first group will include people aged 18 years or older, with more than one long-term health condition who are registered with a GP in the West Midlands County in England that contributes to the Clinical Practice Research Datalink (CPRD) Aurum database. Patients with a terminal diagnosis (prognosis of less than 12 months) will be excluded as well as those deemed by their GP not to have the capacity to consent or participate. For the second group, HCPs currently working in the UK involved in managing patients with multiple health conditions will be eligible to take part. A diverse range of professionals including but not limited to geriatricians, GPs, community pharmacists and specialist nurses will be eligible and invited to participate.

Recruitment

CPRD Aurum is a population-based database of electronic primary healthcare records of anonymised data on diagnoses, tests, prescriptions and demographics,(20) and will be used in the first instance to select eligible patients (Figure 2). Prevalence and incidence data of many conditions in CPRD Aurum have been validated and demographics have been deemed representative of the general population in England.(20) Anonymised patient identifiers of patients from practices within the West Midlands will be accessed using the Data Extraction for Epidemiological Research (DExtER) software.(21) The eligible list of patients' unique CPRD identification numbers will be uploaded to CPRD's interventional research service platform (IRSP). CPRD will contact GP practices asking them to take part in the study. Each practice who takes part will be provided a list of eligible patients together with their CPRD ID number through the IRSP dashboard within their practice. Once this list has been received by the practices, either the practice manager, nurse or GP will view the eligible patients' details. They will review their medical history to ensure they meet the eligibility criteria. Once eligibility is confirmed, these patients will be sent an invitation letter and information sheet (Supplementary material) from their GP. Interested patients will be asked to call or email the OPTIMAL research team, at which point they can ask any questions about the study. Initially 300

For peer review only

eligible patients from 10 GP practices will be selected and invited to participate. Purposive sampling (22) will then be used to ensure maximal diversity in the characteristics of the participants in terms of age, gender, ethnicity, socio-economic status and number and types of long-term health conditions. We aim to recruit up to 30 patients or until data saturation is agreed by the researchers.(23, 24)

HCPs will be recruited through known contacts and British healthcare professional bodies. This will include the British Geriatric Society, Royal College of General Practitioners, Society for Academic Primary Care, Royal College of Physicians, National Pharmacy Association, and the Royal College of Nursing. We will contact these organisations and request they send a letter or email describing the study to all eligible HCPs (Supplementary material). Any HCP interested in taking part will be instructed to email or phone the OPTIMAL research team directly. We aim to recruit until data saturation (24) is reached, up to a maximum of 30 HCPs using purposive sampling based on profession, age, years of experience, sex, ethnicity and place of practice (urban/rural, teaching/non-teaching etc).

Study materials

To encourage a rich dialogue during these interviews, a case vignette (25) (Supplementary material) will be used in conjunction with a semi-structured topic guide (one for patients and one for HCPs; Supplementary material). The topic guides will explore patients' and HCPs' attitudes towards AI-informed clinical decision-making compared to clinician directed decision-making, the perceived advantages and disadvantages of both methods and attitudes to risk management. These topic guides were developed based on the literature and guidance from the OPTIMAL Patient and Public Advisory Group (PPAG).

The case vignettes were co-developed by clinicians within the OPTIMAL team and the PPAG to reflect an expected decision-making process for a patient presenting with a common combination of multiple health conditions with both physical and mental comorbidities. HCPs will be asked how their decision-making process compares to potential outputs of the AI-based tool whereas patients will be asked if the vignette's trajectories fit with their owned lived experiences in terms of the process for receiving their diagnoses and prescriptions. Participants will be encouraged to elaborate on their answers using prompts, probes and follow up questions. The topic guide questions and case vignette were piloted with two patients and one HCP. Following these pilot interviews, the length of the case vignettes was shortened as it was said to be too long during the pilot; no other changes were made.

For peer review only

Data collection

The SSIs will be conducted virtually via Zoom or Teams, by telephone or face-to-face (26, 27) according to participant preference. There will be two interviewers; one will conduct the interview with patients and one will interview HCPs. All interviews will be audio recorded. Age, sex, ethnicity and number of existing conditions will be collected from the patients. Age, sex, ethnicity, profession, and years of experience will be collected from the HCPs who take part. Prior to the interview, all participants will be provided with a summary explanation of AI (Supplementary material) in general and healthcare contexts.

Data analysis

Interviews will be transcribed verbatim and analysed thematically using the Framework Method.(28) NVivo (version 10 for Windows) will be used for data management. The de-identified transcripts generated from the recordings will be passed through word protectors and will be stored with access granted only for the data analysing team, allowing broad perspectives on the data. Codes will be assigned line-by-line and the coding framework derived inductively from the data for both patients and HCPs. Similar codes will be combined and themes will be identified in an analytical framework. After reviewing and revising, the final themes will be determined and the interpretations explored. A summary of overall themes will be sent to patient and HCP participants for comment.

A further analysis of HCP transcripts will be carried out using Buck et al.'s theoretical framework as a starting point to explore and separate out participants' affective, cognitive and behavioural components of their attitudes toward AI use in managing multiple health conditions. Codes will be assigned to Buck et al.'s framework to see how and in what ways the data fits the model and deepens understanding of attitudes toward AI. This framework has not previously been applied to patient perspectives; thus, we do not intend to apply it a priori to the patient transcripts.

As the data coding framework and overall themes emerge these will be discussed with the PPAG and members of the multidisciplinary research team to establish any further areas for exploration or clarification in subsequent interviews. A list of overall and individual themes for patients, and HCPs will be compiled to allow for cross group/individual and purposive sampling characteristics comparison and to understand how individual characteristics of HCPs (e.g. age and prior

For peer review only

experience with AI) and environmental influences (e.g. current working environment, media influences, available technology in the workplace) may influence perspectives.

Patient and public involvement

Members of the public and patients with experience of multiple health conditions have been involved in every stage of the OPTIMAL study. A PPAG was set up at the inception stage of the study comprising eight people who have lived experience of multiple health conditions (either directly or as a carer). We ensured that equality, diversity and inclusivity were prioritised and people of varied age, ethnicity, sex, geographic location in the UK and experience with multiple health conditions were invited to take part. Our PPAG contributed to the development of the study including objective setting, design and study materials through regular meetings. The topic guide, participant information document, consent form and invitation letter for patients were all reviewed by and improved according to feedback from our PPAG before ethical approval was sought. Throughout the study, meetings are expected to take place every three months with the PPAG. It is anticipated that these meetings will be particularly useful during the recruitment, data collection and analyses stages of the qualitative component this protocol describes to ensure the study aims and objectives are being met. To date, the impacts of the PPAG have included: expanding the range of HCPs invited to take part (i.e. to include pharmacists), improved the inclusiveness of the recruitment strategy and amending the design of the case vignettes to be more representative of their experiences.

ETHICS AND DISSEMINATION

Ethical approval for the study was obtained from National Health Service (NHS) Research Ethics Committees (REC) (Reference: 22/SC/0210). Ethical approval to use the anonymised data from the CPRD dataset to select the eligible participants was obtained from the CPRD Expert Review Committee and the Central Advisory Committee (Reference:21_000683).

GPs will not know who decides to participate from the eligible patients they contact for recruitment and likewise, none of the healthcare professional bodies will know which members decide to participate. Written informed consent will be obtained from the face-to face interviews and either electronically completed written consent forms or audio informed consent will be collected from the video or phone interviews; in the latter scenario, researchers will produce a written version of the consent on their behalf. If a participant withdraws from the study within two weeks of the interview,

For peer review only

data collected from the participant will be securely destroyed. Otherwise, all data provided from consented participants will be used in the final analysis.

Data collection began with HCPs in October 2022 and are expected to be completed by September 2023; data collection with patients is expected to begin in August 2023 and completed by November 2023; analyses will be completed by February 2024. The final study report will be circulated to the relevant stakeholders and the summary of the final report will be available to the public by the National Institute for Health and Care Research (NIHR) and accompanied by a plain language summary. Furthermore, the study findings will be shared through peer-reviewed publications, public engagement activities and national or international conferences.

DISCUSSION

Programming an effective and accurate AI-based decision-making tool using advanced machine learning and bioinformatics algorithms is one way to improve our understanding of how to reduce complications and additional comorbidities and safely manage polypharmacy in patients with multiple health conditions. However, patients and HCPs may feel there are barriers, risks and challenges for the use of AI in healthcare as well as benefits. Understanding these perceived barriers and potential risks is vital for effective implementation. This qualitative study will capture patients and HCPs perspectives on an AI-informed decision-making tool developed as part of the OPTIMAL study.

A large proportion of people aged 65 years or older have multiple health conditions.(29) AI-based tools have great potential in optimising disease management in the context of multiple health conditions and polypharmacy where the use of clinical trials are greatly limited. A major strength of this study is the use of data from the CPRD Aurum database to select eligible patients. CPRD Aurum is representative of the UK general population in terms of geographic spread, age, gender, deprivation and patterns of diseases.(30) This will facilitate the selection of a diverse sample of patients with multiple health conditions in terms of known population demographics. Another strength is the study's dedicated PPAG; they have and will continue to play an important role at every stage of the study ensuring that this research and the AI-based tool are of value to patients and carers with multiple health conditions. This also provides a unique opportunity for open dialogue between patients and members of the public, researchers and clinicians so they understand each other's perspectives and can discuss distinct challenges and learn from each other. By conducting qualitative interviews, this

For peer review only

study will provide an in-depth understanding of patient and HCP perspectives which is vital for optimising the development and future implementation of an effective AI tool.(31) The systematic methodology of the Framework Method, input from the PPAG and respondent validation will increase the trustworthiness of the conclusions while reducing researcher bias.(32) A limitation of the study to note is the non-probability sampling and involving patients only from the West Midlands which may limit the generalisability of the findings to patients from other regions in England, however the high socio-demographic diversity (33) of the area will aid understandings about how perceptions may vary between different demographic groups.

To conclude, our study will elucidate the perceptions of HCPs caring for patients with multiple health conditions and patients living with multiple health conditions on the use of AI-based clinical decision support for the management of multiple health conditions. AI-based tools have the potential to improve the care of multiple health conditions through better prescription management and de-prescription; thus, reduce polypharmacy, side-effects, drug burden and ultimately excess morbidity and premature mortality. It is therefore vital for patients and HCPs to raise their concerns and potential barriers regarding the use of an AI-based tool for the tool to effectively work during the shared decision-making process. Our findings will enable a better understanding on how an AI-based tool can be developed and implemented in way that is acceptable to and effectively developed and implemented in a way that is trustworthy and allows effective use by HCPs and is acceptable and accessible to the patient groups who are likely to benefit from the tool the most.

Figure 1. Buck et al.'s model of the general practitioners' determinants of attitudes toward artificial intelligence (AI)–enabled systems. This figure is reproduced under the terms of the Creative Commons Attribution License (https://creativecommons.org/licenses/by/4.0/) from work published in the Journal of Medical Internet Research.(16) Available at: https://www.jmir.org/2022/1/e28916

Figure 2. Flow chart of study participant recruitment

CPRD: Clinical Practice Research Datalink; IRSP: interventional research service platform; OPTIMAL: GP: general practitioners; OPTIMising therapies, disease trajectories, and artificial intelligence (AI) assisted clinical management for patients Living with multiple long-term health conditions

Acknowledgements

The authors would like to thank the members of the PPAG (currently comprised of: Robert Jasper, Christine Michael, Jenny Negus, Gillian Richards, Lynne Wright and Janice Connelly) for their invaluable contribution to the conceptualisation and development of this study thus far.

Author contributions

In collaboration with the PPAG, all authors contributed to the conceptualisation of the study. JC and SF drafted the study tools, led the ethics application and piloted the topic guides and case vignettes with supervision from FC, TAJ, KN and SG. NJG and TEG drafted all versions of the manuscript with supervision from FC. TAJ, KN and SG are senior authors overseeing the project.

Funding source

This work is independent research funded by the National Institute for Health and Care Research (NIHR) (OPTIMising therapies, disease trajectories, and AI assisted clinical management for patients Living with complex multimorbidity (OPTIMAL study), NIHR202632). The views expressed in this publication are those of the author(s) and not necessarily those of NIHR or The Department of Health and Social Care.

Competing Interests

The author(s) declared no potential conflicts of interest with respect to the research, authorship, and/or publication of this article.

For peer review only

References

1. Skou ST, Mair FS, Fortin M, Guthrie B, Nunes BP, Miranda JJ, et al. Multimorbidity. Nature Reviews Disease Primers. 2022;8(1):48.
2. Cassell A, Edwards D, Harshfield A, Rhodes K, Brimicombe J, Payne R, et al. The epidemiology of multimorbidity in primary care: a retrospective cohort study. British Journal of General Practice. 2018;68(669):e245-e51.
3. Johnston MC, Crilly M, Black C, Prescott GJ, Mercer SW. Defining and measuring multimorbidity: a systematic review of systematic reviews. Eur J Public Health. 2019;29(1):182-9.
4. Aoki T, Yamamoto Y, Ikenoue T, Onishi Y, Fukuhara S. Multimorbidity patterns in relation to polypharmacy and dosage frequency: a nationwide, cross-sectional study in a Japanese population. Scientific Reports. 2018;8(1):3806.
5. Schenker Y, Park SY, Jeong K, Pruskowski J, Kavalieratos D, Resick J, et al. Associations Between Polypharmacy, Symptom Burden, and Quality of Life in Patients with Advanced, Life-Limiting Illness. Journal of General Internal Medicine. 2019;34(4):559-66.
6. Jørring Pallesen AV, Kristiansen M, Westendorp RGJ, Mortensen LH. Polypharmacy occurrence and the related risk of premature death among older adults in Denmark: A nationwide register-based cohort study. PLoS One. 2022;17(2):e0264332.
7. Hughes LD, McMurdo ME, Guthrie B. Guidelines for people not for diseases: the challenges of applying UK clinical guidelines to people with multimorbidity. Age Ageing. 2013;42(1):62-9.
8. Bower P, Macdonald W, Harkness E, Gask L, Kendrick T, Valderas JM, et al. Multimorbidity, service organization and clinical decision making in primary care: a qualitative study. Fam Pract. 2011;28(5):579-87.
9. Morrison K. Artificial intelligence and the NHS: a qualitative exploration of the factors influencing adoption. Future Healthc J. 2021;8(3):e648-e54.
10. Young AT, Amara D, Bhattacharya A, Wei ML. Patient and general public attitudes towards clinical artificial intelligence: a mixed methods systematic review. Lancet Digit Health. 2021;3(9):e599-e611.
11. Keel S, Lee PY, Scheetz J, Li Z, Kotowicz MA, MacIsaac RJ, et al. Feasibility and patient acceptability of a novel artificial intelligence-based screening model for diabetic retinopathy at endocrinology outpatient services: a pilot study. Sci Rep. 2018;8(1):4330.
12. Nelson CA, Pérez-Chada LM, Creadore A, Li SJ, Lo K, Manjaly P, et al. Patient Perspectives on the Use of Artificial Intelligence for Skin Cancer Screening: A Qualitative Study. JAMA Dermatol. 2020;156(5):501-12.
13. Scott IA, Carter SM, Coiera E. Exploring stakeholder attitudes towards AI in clinical practice. BMJ Health Care Inform. 2021;28(1).
14. Chang A, editor Common Misconceptions and Future Directions for AI in Medicine: A Physician-Data Scientist Perspective. Artificial Intelligence in Medicine; 2019 2019//; Cham: Springer International Publishing.
15. Reeves S, Albert M, Kuper A, Hodges BD. Why use theories in qualitative research? BMJ. 2008;337.
16. Buck C, Doctor E, Hennrich J, Jöhnk J, Eymann T. General Practitioners' Attitudes Toward Artificial Intelligence-Enabled Systems: Interview Study. J Med Internet Res. 2022;24(1):e28916.
17. Darcel K, Upshaw T, Craig-Neil A, Macklin J, Steele Gray C, Chan TCY, et al. Implementing artificial intelligence in Canadian primary care: Barriers and strategies identified through a national deliberative dialogue. PLOS ONE. 2023;18(2):e0281733.
18. Rosenberg MJ. Attitude Organization and Change; an Analysis of Consistency Among Attitude Components. 1960.
19. Kallio H, Pietilä AM, Johnson M, Kangasniemi M. Systematic methodological review: developing a framework for a qualitative semi-structured interview guide. J Adv Nurs. 2016;72(12):2954-65.

For peer review only

20. Herrett E, Gallagher AM, Bhaskaran K, Forbes H, Mathur R, van Staa T, et al. Data Resource Profile: Clinical Practice Research Datalink (CPRD). Int J Epidemiol. 2015;44(3):827-36.
21. Gokhale KM, Chandan JS, Toulis K, Gkoutos G, Tino P, Nirantharakumar K. Data extraction for epidemiological research (DExtER): a novel tool for automated clinical epidemiology studies. Eur J Epidemiol. 2021;36(2):165-78.
22. Palinkas LA, Horwitz SM, Green CA, Wisdom JP, Duan N, Hoagwood K. Purposeful sampling for qualitative data collection and analysis in mixed method implementation research. Adm Policy Ment Health. 2015;42:533-44.
23. Patton MQ. Enhancing the quality and credibility of qualitative analysis. Health Serv Res. 1999;34(5 Pt 2):1189-208.
24. Morse JM. Determining Sample Size. Qualitative Health Research. 2000;10(1):3-5.
25. Tremblay D, Turcotte A, Touati N, Poder TG, Kilpatrick K, Bilodeau K, et al. Development and use of research vignettes to collect qualitative data from healthcare professionals: A scoping review. BMJ Open. 2022;12(1):e057095.
26. Keen S, Lomeli-Rodriguez M, Joffe H. From challenge to opportunity: virtual qualitative research during COVID-19 and beyond. International journal of qualitative methods. 2022;21:16094069221105075.
27. Sturges JE, Hanrahan KJ. Comparing telephone and face-to-face qualitative interviewing: a research note. Qual Res. 2004;4(1):107-18.
28. Gale NK, Heath G, Cameron E, Rashid S, Redwood S. Using the framework method for the analysis of qualitative data in multi-disciplinary health research. BMC Med Res Methodol. 2013;13:117.
29. Barnett K, Mercer SW, Norbury M, Watt G, Wyke S, Guthrie B. Epidemiology of multimorbidity and implications for health care, research, and medical education: a cross-sectional study. Lancet. 2012;380(9836):37-43.
30. Wolf A, Dedman D, Campbell J, Booth H, Lunn D, Chapman J, et al. Data resource profile: Clinical Practice Research Datalink (CPRD) Aurum. Int J Epidemiol. 2019;48(6):1740-g.
31. Hogg HDJ, Al-Zubaidy M, Talks J, Denniston AK, Kelly CJ, Malawana J, et al. Stakeholder Perspectives of Clinical Artificial Intelligence Implementation: Systematic Review of Qualitative Evidence. J Med Internet Res. 2023;25:e39742.
32. Birt L, Scott S, Cavers D, Campbell C, Walter F. Member Checking: A Tool to Enhance Trustworthiness or Merely a Nod to Validation? Qual Health Res. 2016;26(13):1802-11.
33. Patel K NA. Diversity and cultural leadership in the West Midlands. A report for the West Midlands leadership commission. 2018 March.

For peer review only

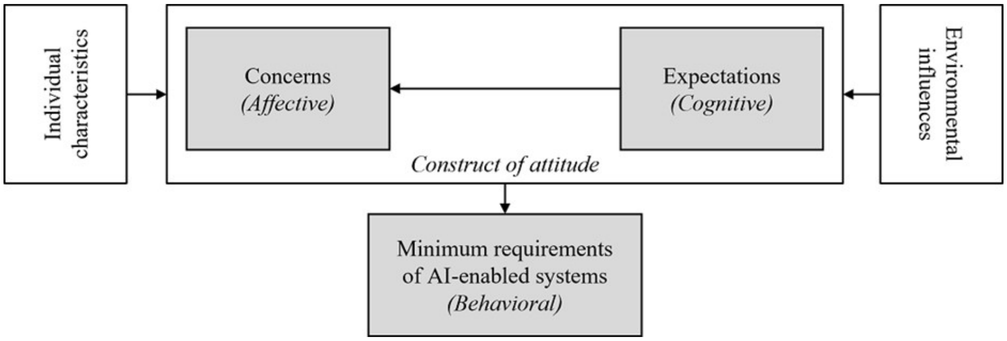

Figure 1. Buck et al.'s model of the general practitioners' determinants of attitudes toward artificial intelligence (AI)–enabled systems.

165x55mm (144 x 144 DPI)

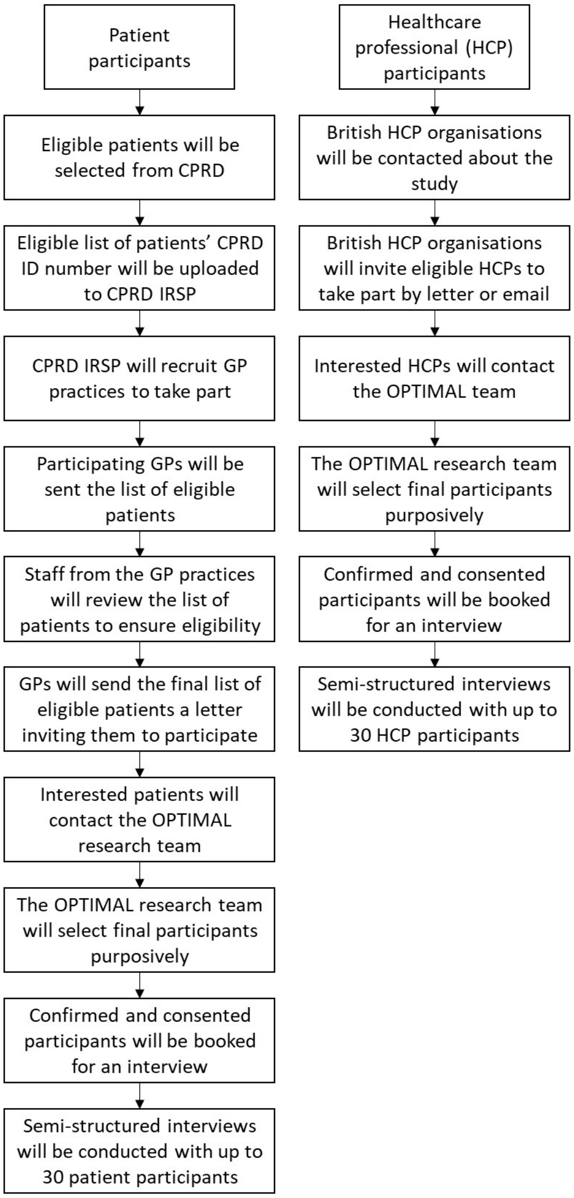

Flow chart of study participant recruitment

132x277mm (150 x 150 DPI)

Supplementary Material 1

Information sheet/ Invitation letter

Practice address/contact details

<<Patient Address>>

<<Date>>

OPTIMIsing therapies, disease trajectories, and AI assisted clinical management for patients Living with complex multimorbidity (OPTIMAL study)

Your Unique Reference Code is: CPRD *<<Global ID>>*

Dear *<<Patient name>>*

We would like to invite you take part in a research study. The research team are hoping to speak to people who have multiple (four or more) long-term health conditions to discuss their thoughts about how artificial intelligence (AI) may help them and their health care professionals manage these conditions. They will also be speaking to health care professionals to help understand their views.

Before you decide whether you would like to take part in an interview, please read the enclosed participant information sheet that explains the study in more detail and what would happen if you agree to take part.

If you would like to participate in an interview, please contact a member of the research team whose details are given below.

The research team:
OPTIMAL Team
Institute of Allied Health Research, University of Birmingham, B15 2TT

For more information, contact:
[name, email and contact number of study lead]

Thank you for taking the time to consider taking part in the study.

Yours sincerely,

<<GP sign off>>

For peer review only

UNIVERSITY OF BIRMINGHAM

NHS National Institute for Health Research

OPTIMIsing therapies, disease trajectories, and AI assisted clinical management for patients Living with complex multimorbidity (OPTIMAL)

For peer review only

Participant information sheet: patient interviews

Introduction

We would like to invite you to take part in a research study. Before you decide to take part, we would like to explain why we are doing this study and what your involvement will mean for you. Please take time to read this information sheet and discuss it with others if you wish. Ask us if there is anything that is not clear or if you would like more information before deciding whether or not to take part. Our contact details are at the end of this information sheet.

What is the study about?

The study aims to use artificial intelligence (AI) to produce computer programmes and tools that will help improve the treatment and choice of medications in patients with clusters of multiple long-term conditions (we say these people have complex multimorbidity or cMM). Artificial intelligence (or AI) is a computer system that can conduct tasks that would normally need human intelligence. There are examples of AI being used in our everyday lives, with applications and software such as Spotify, Amazon and BBC iplayer. These 'apps' and on-line websites function by predicting what kind of music, TV programmes, or general purchases we may like or want. They do this by using a computer programme that can observe what categories and kinds of TV, Music etc. we watched or bought in the past and use this data to predict what we are a likely to choose in the future.

The machine learning branch of AI is also used in things like self-driving cars, language translations and it is being developed for use in healthcare. Newer AI methods makes it easier to process large amount of health data in a short time. These AI methods can give doctors and patients information that may help improve the care of people with multiple long-term health conditions

AI can be used in healthcare to help guide the diagnosis of long-term health conditions, plan the best treatment strategies, and predict the next health condition that people might develop. This is especially relevant for people who have several different health conditions

because the guidelines that doctors and healthcare professionals may use for one condition does not routinely consider other health conditions.

AI-based decision-making tools (computer programs) for managing multiple health conditions may help patients and healthcare professionals make more well-informed shared decisions. Patients and healthcare professionals may see many benefits to the use of AI in healthcare. However, they may be concerned this could be harmful or that it could affect the relationships between healthcare professionals and patients. We want to speak to patients and healthcare professionals to understand their views about AI in healthcare. It is important in healthcare research that we reflect these experiences in any future plans for the use of AI in a healthcare setting.

Data collected from this study will give new insights to how healthcare professionals and people with multiple long-term health conditions view AI-based decision-making tools. It will also tell us which factors in the computer program are important to them and what options they prefer. This information will also be used to help to develop how these tools are used in clinical practice.

How will we do this?

1. By using artificial intelligence (AI) methods with electronic health records to generate data models that tell us how the different mixes of conditions arise over time and how certain drugs can make this better or worse.

2. By asking people with cMM and doctors about their knowledge and views about using AI to make decisions about health care.

3. By using AI computer techniques to combine data, and together with the input from people with cMM and doctors, develop a computer program. This will predict which drug we should give and when we should give it to someone with cMM to reduce risk and bring about maximum benefit. It will also tell us what disease people may get next. These predictions will be based upon data gathered from a large number of patients who have similar conditions and prescribed medication.

4. By examining the best way to present information in the AI tool to people with cMM and doctors by asking them about what is important to them and what options they prefer.

Our team includes patients, public members, and world leading experts from universities with expertise in biology, AI, medicine, health service research, public health, and general practice. Working as a multidisciplinary team we hope to improve the health and care of people with cMM.

Why have I been asked?

You have been invited because we would like to talk to you about your experiences living with four or more long-term health conditions.

Do I have to take part?

For peer review only

Taking part in this study is completely optional, the decision about whether to take part is up to you. Whether you decide to take part or not will have no effect upon your healthcare and treatment and you can leave the study at any time, without giving a reason.

What will happen to me if I take part?

If you choose to take part, you can contact the research team by telephone or email (details below). The researcher can provide more information about the study, and answer any questions you may have.
Please note that because we would like to interview a range of people depending upon certain characteristics, we will ask you for some basic details including your age, ethnicity, gender and number of health conditions). It may take a number of weeks before we can to arrange an interview date. In the event that we do not require your assistance, we will contact you to let you know.
The interviews will take place either face-to-face or remotely using videoconferencing or telephone at a time convenient to you. The interview should last for around 60 minutes, although you can ask for a break or stop the interview at any time.

The interviewer will ask questions about your thoughts on how AI informed decision making (the use of computer programmes) compares with doctors making decisions about your health.

We will also show you an example of a patient with several long-term conditions and how their health care has been managed using a computer programme, for example, how the computer programme has helped decide what medications they may need. We will ask you what kinds of things are important to know if a computer is informing these kinds of decisions about the medications you are prescribed your health in general.

If you agree to take part, we would like to audio record the interview using a secure recording device. After the interview, the recording will be written down (in a transcript) but all names and place names will be removed and no comments will be linked to you or any other person. The recording and transcript will be kept completely confidential using a study ID code and only the University evaluation team will have access to them.

At the end of the interview, you will be asked if you would be willing to be contacted at a later date to consider involvement in the second stage of the study. This stage will aim to explore preferences in terms of how computer programmes for AI directed clinical decision making are presented. For example, how the computer displays information, and what things patient think are important to see.

What are the possible benefits of taking part?

Although there is no direct benefit to you if you take part in an interview, the interviews will give us important information that should help patients with cMM along with the doctors who manage their conditions.

For peer review only

Will I receive any financial reimbursement for taking part?

You will be offered a £15 voucher (Amazon or Love 2 Shop) as a thank you for taking part in the interview.

What are the possible risks and disadvantages of taking part?

It may be upsetting to talk about living with multiple long-term conditions and your health care experiences. If this happens, we can stop or pause the interview at any time. We have put together a list of organisations that you can get in contact with who may be able to help. Many people have said that they find it helpful to talk to researchers about their experiences of living with conditions.
Taking part in the interview will take some of your time. However, you can choose a time and date that is suitable for you.
In light of COVID-19, we will adhere to current government guidelines and local procedures in order to minimise the risk of exposure. Support for participants will be provided to set up an online conferencing link, or by telephone according to participant preference. Researchers will have evidence of vaccination and/or negative results from a lateral flow test prior to interview if face-to-face.

What if there is a problem?

If you have any concerns, please speak to a member of the research team in the first instance, you can get in touch with us using the contact details at the end of this information sheet. We will do our best to answer your questions. If your concerns are not addressed and you wish to make a formal complaint, you can refer to the Patient Liaison Services (PALS) 0121 424 0808 PALS@uhb.nhs.uk.

Will my taking part in the study be kept confidential?

All information collected about you for this study will be subject to the General Data Protection Regulation and Data Protection Act 2018 for health and social care research and will be kept strictly confidential.

All audio-recordings will be kept for 10 years after the end of the study and then destroyed. Documentation and data from this part of the study will be securely stored at the University of Birmingham for 10 years.

How will we use information about you?

The University of Birmingham is the Sponsor for this study and this means that the University of Birmingham are responsible for looking after your information and using it properly. University of Birmingham and the NHS will keep identifiable information about you for at least 10 years after the study has finished, to allow the results of the study to be verified if needed. Information collected from you will include:

- Name
- Age

For peer review only

- Ethnicity
- Gender
- Number of long-term conditions

People who do not need to know who you are will not be able to see your name or contact details. Your data will have a unique study number instead and all information will be kept safe and secure. In the research team, you will be identified using your unique study number.

All information collected by the Sponsor, including a copy of your signed consent form, will be securely stored at the research study office at the University of Birmingham on paper and electronically and will only be accessible by authorised personnel. The only people in the University of Birmingham who will have access to information that identifies you will be people who manage the study or audit the data collection process.

The audio recordings from the interview will be transcribed by a transcription company which has been approved for transcription of medical data. If you agree to take part in the interview study, your name will not be on the recording and we will remove your name from the interview transcripts to keep your identity confidential. Direct quotes may be used in publications but these will be numbered and anything that could identify you will be removed. Nothing that you say will be fed back to the doctors and nurses involved in your care as coming from you.

What are your choices about how your information is used?

You can choose to stop taking part in the study at any time, without giving a reason, but we will keep information about you that we already have. If you agree to take part in this study, you will have the option to take part in future research using your data saved from this study. To safeguard your rights, we will use the minimum personally identifiable information possible. You can find out more about how your information will be used at https://www.birmingham.ac.uk/privacy/index.aspx.

The NHS and the University of Birmingham will use your name and contact details to contact you about the research study, and make sure that relevant information about the study is recorded and to oversee the quality of the study. Individuals from the University of Birmingham and regulatory organisations may look at your research records to check the accuracy of the research study.

All individuals who have access to your information have a duty of confidentiality to you. Under the provisions of the General Data Protection Regulation (GDPR) 2018, you have the right to know what information the Trial Office has recorded about you. If you wish to view this information, or find more about how we use this information, please contact the University of Birmingham's Data Protection Officer at the address below.

Where can you find out more about how your information is used?

For peer review only

If you would like more information on your rights, would like to exercise any right or have any queries relating to our processing of your personal data, or if you wish to make a complaint about how your data is being or has been processed, please contact:

The Data Protection Officer, Legal Services, The University of Birmingham, Edgbaston, Birmingham B15 2TT
Email: dataprotection@contacts.bham.ac.uk
Telephone: +44 (0)121 414 3916

You can also find out more from www.hra.nhs.uk/information-about-patients/ and by reading the information available here **www.hra.nhs.uk/patientdataandresearch.**

What will happen if I don't want to carry on with the study?

Your participation is completely voluntary. If you choose to take part but change your mind later, you are free to leave the study at any time, without giving a reason, and without your healthcare being affected. If you wish to withdraw from the study, please contact a member of the team (details are at the end of this document), However, please note that if you decide to withdraw more than 2 weeks after participating in an interview, any data already collected may still be used in the study.

What will happen to the results of the research study?

The information we collect will be analysed and the results will be presented in several ways:

- A short written summary of the results will be available on the OPTIMAL website hosted by the University of Birmingham, or can be sent out to participants should they prefer.
- A detailed report will be written and will be available for participants on the OPTIMAL website hosted by the University of Birmingham, or can be sent out to participants should they prefer.
- We will publish the results in academic journals.

Your details will not be shared at any time and you will not be identified in any of the results from the research.

Who is organising and funding the research?

The research study is funded by the National Institute for Health Research (NIHR). The research is sponsored and insured by the University of Birmingham.

Who has reviewed the study?

All research in the NHS is looked at by an independent group of people called a Research Ethics Committee (REC), to protect your interests. This study has been reviewed and given favourable opinion by South Central – Hampshire B Research Ethics Committee (REC

For peer review only

Reference: 22/SC/0210). Patients and public Involvement (PPI) members have been involved throughout all stages of the research study.

What happens next?

If you would like to participate in an interview, please contact a member of the research team whose details are given below.

The research team:
OPTIMAL Team
Institute of Allied Health Research,
University of Birmingham
B15 2TT

For more information, contact:
[name, email and contact number for study lead]

For peer review only

UNIVERSITY OF BIRMINGHAM

NIHR | National Institute for Health and Care Research

Supplementary Material 2

Study Title: **OPTIMIsing therapies, disease trajectories, and AI assisted clinical management for patients Living with complex multimorbidity (OPTIMAL study)**

Dear Colleague

We are writing to you on behalf of a team of researchers based at the University of Birmingham who are undertaking the above named study which aims to use artificial intelligence (AI) to produce computer programmes and tools that will help improve the treatment and choice of drugs in patients with clusters of multiple long-term conditions (complex multimorbidity or cMM).

As part of this study, the team are hoping to speak to healthcare professionals who care for patients with multiple (or more than four) long-term conditions (complex multimorbidity) to discuss their thoughts about how artificial intelligence (AI) may help professionals and patients manage these conditions. We will also be speaking to patients with long-term conditions to get their views.

We have attached a copy of the participant information sheet (PIS), to provide more details about the study. Before you decide whether you would like to take part in an interview, please read the enclosed participant information sheet that explains the study in more detail and what would happen if you agree to take part. If this is something you may be interested in taking part in, please email the address below.

Yours sincerely,

[name, email and contact number of study lead]

Institute of Applied Health Research
University of Birmingham
B15 2TT

For peer review only

Supplementary material 3

Case study for participants with multiple health conditions

Harinderjeet is 53 years old and owns a plumbing business with several employees. Harinderjeet has asthma, chronic lung disease, migraine, osteoarthritis (joint pain) and angina (chest pain). His work is very important to him as he feels responsible for his employees and wants to develop the business further. Most notably, his work helps to keep him distracted from this health conditions and pain.

He was diagnosed with asthma when he was 42 years, angina and osteoarthritis at age 45 years and then with lung disease two years later. As a result of his diagnoses he gave up smoking and has been smoke-free for the past five years. His GP told him he should exercise more and he tries to but the pain he experiences in his joints makes it difficult and he hardly has the time with running his business. When he last saw his GP last week he had his weight measured and his BMI was 29 kg/m^2 and his doctor said that he should think about losing some weight. Harinderjeet thinks that the worsening of some of his symptoms and health conditions are caused by side effects of some of the drugs he takes; he takes approximately 20 tablets a day.

Harinderjeet spends a large amount of time going to doctor and hospital appointments, both planned and unplanned contacts with the health care system about once a week. His is mainly in contact with his GP who will regularly review the drugs for his pain and asthma but then has separate appointments at the hospital for his lung disease and arthritis. He also sees a clinical nurse specialist for his angina. He started developing some severe pain in his tummy and told the doctor at the arthritis clinic who told him that it wasn't related to his arthritis and he should talk to his GP about it.

Q1. How does Harinderjeet's experience of being diagnosed with multiple health conditions compare with your experience? Explain some of the similarities and the differences.

Q2. How does Harinderjeet's experience of feeling that some of the worsening of his symptoms and being diagnosed with other health conditions are due to the medicines he is taking compare with your experience? Explain some of the similarities and the differences.

For peer review only

Case vignette for healthcare professionals - Janet

Janet is a white English woman aged 72 years who is a retired secretary living at home on her own.

From her records you can see:

She had an NSTEMI 3 years ago.

She has hypertension diagnosed over 10 years ago, type 2 diabetes diagnosed 6 years ago, and she has chronic kidney disease stage 3a.

BMI - 32.2 kg/m2.
Average home blood pressure is 134/82.
Renal function is stable with an eGFR of 55.
Diabetes is stable and reasonably well controlled with a Hba1c of 52.

She is a non-smoker and drinks occasionally. She does minimal exercise.

Her medications are:

Ramipril 10mg - ischaemic heart disease, CKD, diabetes, hypertension

Amlodipine -5mg hypertension

Glyceryl trinitrate spray - as required. - ischaemic heart disease.

Aspirin 75mg - ischaemic heart disease

Bisoprolol 10mg - ischaemic heart disease, hypertension

Atorvastatin 80 mg - ischaemic heart disease

Metformin - modified release 500mg BD - diabetes.

In today's consultation:

Her husband died 10 years ago, and she has a supportive family who live nearby but struggles with loneliness, especially at night time. She had bereavement counselling at the time of his death which helped her a lot. She used to go to church regularly until about 6 months ago and

For peer review only

has been a member of a choir for many years. However, over the past 6 months she has lost interest in going to these things any more (or joining the online versions even though she knows how). She has very poor sleep, reduced appetite, she can't concentrate on a book or TV programme. She feels quite "empty" and tired a lot of the time. She often thinks it might be better if she did not wake up the next day, but she does not have any plans for suicide. **You have diagnosed her with moderate to severe depression and she is now asking whether she could try some medication.** She knows her son has has been taking fluoxetine for many years for his depression and he found this really helpful. She is also willing to try some CBT.

For peer review only

Q1: What factors in the history (demographics, symptoms, past medical history, medications) would you consider when deciding how to manage her depression? (pt's age, sleep/appetite problems, other health problems, other medications, patient preference)

Q2: What factors or resources would you normally use to help make complex decisions like this? (own experience, NICE guidance, other guidance, patient preference?)

Q3: Assuming you decide to start an antidepressant, what would you recommend for Janet, and why?

Q4: The computer algorithm says Janet should take sertraline with a PPI, do you agree? (this is just an example, and I don't know if that would be the answer and whether it will give an explanation or not)

Q5: How would you feel about using the computer algorithm to make a decision like this compared with your usual practice? If you agree with the algorithm/ if you don't agree?

Very comfortable   Mostly comfortable   Not sure   Mostly uncomfortable   Very uncomfortable

Why?

Relevant NICE guidelines copied below:

From NICE guidelines; https://cks.nice.org.uk/topics/depression/management/new-or-initial-management/

Which antidepressants are recommended for people with chronic physical health problems?

- Prescribe a generic selective serotonin reuptake inhibitor (SSRI), such as sertraline or citalopram unless these are contraindicated or there is a significant drug interaction.
    - Do not use citalopram or escitalopram in people who are taking medication that could prolong the QT interval.
- For a brief summary on which antidepressants may be prescribed for people with chronic physical health problems, see Table 1. For more detailed information see appendix 16 of the full NICE guideline.

Table 1. Antidepressants recommended for people with a chronic physical health problem.

Medication being taken for a chronic physical health problem	Antidepressants that should not normally be offered	Suitable options
Nonsteroidal anti-inflammatory drugs (NSAIDs)	Selective serotonin reuptake inhibitor (SSRI) or a serotonin noradrenaline reuptake inhibitor (SNRI) (if no suitable alternative can be found, offer gastroprotection)	Mirtazapine, moclobemide, reboxetine, or trazodone
Aspirin	SSRIs and SNRIs (if no suitable alternative can be found, offer gastroprotection)	Mirtazapine.<br>When aspirin is used alone, consider trazadone or reboxetine.

Supplementary material 4

OPTIMIsing therapies, disease trajectories, and AI assisted clinical management for patients Living with complex multimorbidity (OPTIMAL study)

Interview guide: Patient Interview

This is the starting topic guide. The overarching objectives will remain the same, but questions and prompts will be developed as interviews are undertaken to incorporate any important themes emerging from the interviews.

Before the interview begins

- Ensure the participant has read the information leaflet
- Ensure the participant has had the opportunity to ask any questions about the research including issues about confidentiality, the findings of the research and where the research will be disseminated before being asked to agree to each item on the consent form.
- Start audio-recording
- Go through each item on the consent form and record their verbal consent. Explain that you will send/email a copy of the consent form for their records. They should already have a copy of the participant information sheet with details about the study, how to withdraw etc
- Explain that they don't have to answer all the questions just because they have consented to the interview, and that they can take a break or stop the interview at any time.
- Explain that you are there to understand more about their experiences and that they will have some time at the end of the interview to talk about any other issues that are important to them that may not have been covered by the questions.
- Check that they are happy to continue to be audio-recorded.
- Begin the interview.

TOPICS TO BE COVERED IN THE INTERVIEW

<u>Patient's reflections on living with/managing long-term conditions</u>

Could you tell me about your health conditions? Prompts – how long have you had conditions, how have they developed over time

What are your experiences of managing patients your long-term conditions? Prompts – medication, self-management, navigating primary/specialist care, relationships with clinicians/HCPs

Have you experienced any difficulties managing your conditions? Prompts -

- Side-effects from medication (impact on appetite, understanding which medications are for which condition; remembering to take medication)
- Multiple appointments for different conditions; opportunities to see GP/specialists; information sharing;

For peer review only

- Impact of conditions upon other activities e.g. work, time to socialise, leisure activities, access
- Impact upon relationships

<u>Acceptability of AI in clinical decision making</u>

Attitudes and understanding about AI in general terms (briefing guide of discussion points about AI)

Have you any thoughts about AI and how it is used in everyday activities?
Do you have any experience of AI being used to manage your healthcare?

<u>Attitudes towards and perspectives about AI health care in managing long term conditions</u>

In this study interview we want to explore the advantages and limitations of AI directed clinical decision making and compare this with doctors making decisions.

How do you feel about how clinical decisions are made about your healthcare? E.g. do you feel involved in decisions?

Do you think the use of AI (computer programs) in healthcare decision making could be beneficial? If so, how could it be used? If not, what are the reasons why?

Do you think that AI (computer programs) could improve clinical decision making?

Do you think that AI (computer programs) could help you manage your health conditions? If so, how may this benefit you and what would 'good' look like?

In which situations do you think AI (computer programs) should not be used?

What would your concerns be, if any?

How do you feel that using AI (computer programs) for clinical decision making may compare to clinician/patient making decisions about your health?

Can you think of ways AI (computer programs) making decisions about your care may be preferable to clinicians making decisions about your care?

Can you think of ways clinician/patient guided decisions about your care are preferable to AI directed decisions about your care? E.g. continuity of relationship; importance of relationship with HCP

What kind of questions might you want to ask before agreeing to treatment that was directed by AI?

What kind of things may influence your choice between AI or clinician/patient guided decisions about your care?

How confident would you feel if prescription decisions were directed by AI (computer program/predictive algorithm) rather than your doctor?
Very comfortable   Mostly comfortable Not sure   Mostly uncomfortable Very uncomfortable

For peer review only

Why?

Stage two: Vignettes – validity of AI in clinical decision making

We are developing a tool that will detect what other conditions a patient may develop in the future based upon their current health conditions. It will also provide information about what sort of medications should be prescribed (to achieve maximum health benefits and reduce side-effects etc)

Participants will have been sent a copy of the case vignette prior to the interview

We will present to the participant, a simulated patients disease clusters, to compare how aspects of the patient care management fit in with their own experiences:
The case vignette will present a simulated patients disease clusters, with 4 health conditions showing:

- How conditions developed
- What other conditions the patient developed
- Impacts of conditions upon their lifestyle and relationships etc
- How medications were managed
- How overall health management is navigated via the AI algorithm

How does the patient's experience of being diagnosed with multiple health conditions compare with your experience? Explain some of the similarities and the differences.

How does the patient's experience of feeling that some of the worsening of their symptoms and being diagnosed with other health conditions are due to the medicines he is taking compare with your experience? Explain some of the similarities and the differences.

What other things would you want to know about this patients to inform decisions about management of their treatment e.g lifestyle factors?

Would it be useful to know for example what conditions may develop in the future?

Would this impact upon things like lifestyle changes? E.g. dietary/exercise/self-monitoring regime

How would you feel about a computer programme predicting the medications you may benefit most from rather than your doctor?

Very comfortable   Mostly comfortable   Not sure   Mostly uncomfortable   Very uncomfortable

Does this case simulation reflect your own kind of experiences and preferences in terms of how your cMM are managed?

What do you think are the most important things that researchers need to consider when developing computer programmes to manage patient care?

Conclusion of interview

For peer review only

Now we've talked a bit about how AI could be used, how do you feel about it?

Thinking back to the difficulties you talked about in managing treatments, do you think AI could have an effect on this?

Thank you. That was my last question.

Is there anything you would like to add about the things we talked about but have not covered in the interview?

Any questions from interviewee

Reminder of study contact details and signposting

For peer review only

NHS National Institute for Health Research

UNIVERSITY OF BIRMINGHAM

OPTIMIsing therapies, disease trajectories, and AI assisted clinical management for patients Living with complex multimorbidity (OPTIMAL):

Interview guide: Staff Interview

This is the starting topic guide. The overarching objectives will remain the same, but questions and prompts will be developed as interviews are undertaken to incorporate any important themes emerging from the interviews.

Before the interview begins

- Ensure the participant has read the information leaflet
- Ensure the participant has had the opportunity to ask any questions about the research including issues about confidentiality, the findings of the research and where the research will be disseminated before being asked to agree to each item on the consent form.
- Start audio-recording
- Go through each item on the consent form and record their verbal consent. Explain that you will send/email a copy of the consent form for their records. They should already have a copy of the participant information sheet with details about the study, how to withdraw etc
- Explain that they don't have to answer all the questions just because they have consented to the interview, and that they can take a break or stop the interview at any time.
- Explain that you are there to understand more about their experiences and that they will have some time at the end of the interview to talk about any other issues that are important to them that may not have been covered by the questions.
- Check that they are happy to continue to be audio-recorded.
- Begin the interview.

TOPICS TO BE COVERED IN THE INTERVIEW

<u>Managing patients with four or more long-term conditions</u>?

What are your experiences of managing patients with four or more long-term conditions? Prompt – what factors are important to consider when compared with patients with single diseases , comorbidties, other meds

For peer review only

What difficulties do patients with four or more long-term conditions have in managing their conditions and making decisions about treatment options?

Acceptability of AI in clinical decision making

Attitudes and understanding about AI in general terms (briefing guide of discussion points about AI) such as Amazon/ BBC iPlayer/ Spotify giving recommendations based on the existing data they have collected?

Do you have any experience of using AI in clinical practice?

Attitudes towards and perspectives about AI health care in managing cMM.

How do you think using AI directed clinical decision making may compare to usual practice?

Thinking about the impact on health care professionals ***and*** patients:

Thinking specifically about managing physical health conditions: Prompt? Diabetes, COPD, heart failure, CKD:

What are your thoughts on advantages and disadvantages of AI directed clinical decision making compared with your usual practice?

How do you think patients feel about AI directed clinical decision making?

What do you think patients may see as the advantages/ disadvantages of AI directed clinical decision making?

Are there examples where either using AI or using usual practice might be preferable?

What kind of things may influence your choice?

What are your thoughts on using AI directed clinical decision making with patients who have mental health conditions, e.g. depression/ anxiety?

What are your thoughts about using AI directed clinical decision making to make decisions about prescribing in physical health conditions (e.g., choosing the best medication for diabetes).

What are your thoughts about using AI directed clinical decision making to make decisions about prescribing treating mental health conditions (e.g., choosing the best antidepressant).

How confident would you feel if prescription decisions were directed by a predictive algorithm compared to your usual practice?

Very comfortable Mostly comfortable Not sure Mostly uncomfortable Very uncomfortable

For peer review only

Why?

Stage two: Vignettes – validity of AI in clinical decision making

We will present to the participant, a range of simulated patients from a range of disease clusters, to compare how aspects of the AI patient care management fit in with their own experiences a clinician:

What resources would you normally use to help make complex decisions like this? (NICE guidelines, own experience, other guidelines)

What medication changes would you recommend and why? (pt's age, sleep/appetite problems, other health problems, other medications, patient preference)

The computer algorithm says the patient should take X with the following explanation**, do you agree?**

How would you feel about using the computer algorithm to make a decision like this compared with your usual practice?

Very comfortable Mostly comfortable Not sure Mostly uncomfortable Very uncomfortable

Why?

How would you feel if the algorithm recommended a treatment you were not expecting? Would you over-ride if you disagree?

What would you do if a patient didn't want to follow the recommended treatment (i.e., would you look to over-rule it or stick by it?

Close of interview

Thank you. That was my last question. Is there anything you would like to add about the things we talked about but have not covered in the interview?

For peer review only

Supplementary materials 5

Artificial Intelligence (AI) Background for participants

Artificial Intelligence or AI is the term used to describe a computer system or algorithm that can conduct tasks that would normally require human intelligence.

There are examples of AI being used in our everyday lives, with applications and software such as Spotify, Amazon and BBC iPlayer. These 'apps' and on-line websites function by predicting what kind of music, TV programmes, or general purchases we may like or want. They do this by using a computer programme that can observe what categories and kinds of TV, music etc. we watched or bought in the past and use this data to predict what we are likely to choose in the future.

AI is also used in things like self-driving cars and language translations, and it is also being increasingly developed for use in healthcare. For example, AI has been developed that can screen the retina scans of patients with suspected diabetic eye disease. This is the leading cause of blindness in adults, and in many parts of the world, there are not enough doctors or health care professionals to undertake the work involved in diagnosing the condition. The AI trained system can diagnose the condition with the same accuracy as a trained healthcare professional.

In healthcare AI is also being developed to help us to predict how patients' health may progress in the future. It can also help to decide what are the best treatments and medications for the increasing number of people who are living with several long-term health conditions

Using the large amounts of anonymised patients' data available from GP and hospital electronic health records, AI can help us predict the life trajectories of people with multiple long-term conditions. For example, we know that people with diabetes may already have or are more likely to develop high blood pressure, heart disease and eye disease. These patients are usually prescribed medications to help manage all these conditions, but it can be difficult to ensure the best medications are prescribed for each individual patient based on the complexities of their own specific medical history, other characteristics and the variability in decisions made by different health professionals providing care.

Given the variability of factors that can influence health, having an efficient, accurate and easy to use AI programme that can take into account this variability and help 'predict' the best combination of medications, or what conditions may develop in the future may benefit patients and health care professionals.

For peer review only

BMJ Open

Perceptions on artificial intelligence-based decision making for coexisting multiple long-term health conditions: protocol for a qualitative study with patients and healthcare professionals

Journal:	*BMJ Open*
Manuscript ID	bmjopen-2023-077156.R1
Article Type:	Protocol
Date Submitted by the Author:	26-Oct-2023
Complete List of Authors:	Gunathilaka, Niluka; Ministry of Health Sri Lanka,<br>Gooden, Tiffany; University of Birmingham College of Medical and Dental Sciences,<br>Cooper, Jennifer; University of Birmingham, Institute of Applied Health Research<br>Flanagan, Sarah; University of Birmingham, Institute of Applied Health Research<br>Marshall, Tom; University of Birmingham, Public Health and Epidemiology<br>Haroon, Shamil; University of Birmingham,<br>D'Elia, Alexander ; University of Birmingham College of Medical and Dental Sciences<br>Crowe, Francesca; University of Birmingham, Institute of Applied Health Research<br>Jackson , T; University of Birmingham,<br>Nirantharakumar, Krishnarajah; University of Birmingham, Institute of Applied Health Research<br>Greenfield, Sheila; University of Birmingham, Institute of Applied Health Research
**Primary Subject Heading**:	Public health
Secondary Subject Heading:	Qualitative research
Keywords:	QUALITATIVE RESEARCH, Primary Health Care, PUBLIC HEALTH

SCHOLARONE™ Manuscripts

Perceptions on artificial intelligence-based decision making for coexisting multiple long-term health conditions: protocol for a qualitative study with patients and healthcare professionals

Niluka J Gunathilaka[1*], Tiffany E Gooden[1*], Jennifer Cooper[1], Sarah Flanagan[1], Tom Marshall[1], Shamil Haroon[1], Alexander D'Elia[1], Francesca Crowe[1], Thomas A Jackson[2,3], Krishnarajah Nirantharakumar[1], Sheila Greenfield[1]

1. Institute of Applied Health Research, University of Birmingham, Birmingham, UK
2. Institute of Inflammation and Ageing, University of Birmingham, Birmingham, UK
3. Department of Healthcare for Older People, Queen Elizabeth Hospital, Birmingham, UK

* Joint first authors

Correspondence to:

Francesca Crowe

Institute of Applied Health Research, University of Birmingham, Birmingham, UK

f.crowe@bham.ac.uk

Keywords: multimorbidity; patient perspectives, clinical support, artificial intelligence, qualitative methods, shared decision-making

Word count: 2942

For peer review only - http://bmjopen.bmj.com/site/about/guidelines.xhtml

ABSTRACT

Introduction

Coexisting multiple health conditions is common among older people, a population that is increasing globally. The potential for polypharmacy, adverse events, drug interactions and causing additional health conditions complicates prescribing decisions for these patients. Artificial Intelligence (AI)-generated decision-making tools may help guide clinical decisions in the context of multiple health conditions, by determining which of multiple medication options is best. This study aims to explore the perceptions of healthcare professionals (HCPs) and patients on the use of AI in the management of multiple health conditions.

Methods and analysis

A qualitative study will be conducted using semi-structured interviews. Adults (≥18 years) with multiple health conditions living in the West Midlands of England and HCPs with experience in caring for patients with multiple health conditions will be eligible and purposively sampled. Patients will be identified from Clinical Practice Research Datalink (CPRD) Aurum; CPRD will contact general practitioners who will in turn, send a letter to patients inviting them to take part. Eligible HCPs will be recruited through British healthcare professional bodies and known contacts. Up to 30 patients and 30 HCPs will be recruited, until data saturation is achieved. Interviews will be in-person or virtual, audio recorded and transcribed verbatim. The topic guide was designed to explore participants' attitudes towards AI-informed clinical decision-making to augment clinician-directed decision-making, the perceived advantages and disadvantages of both methods and attitudes toward risk management. Case vignettes comprising a common decision pathway for patients with multiple health conditions will be presented during each interview to invite participants' opinions on how their experiences compare. Data will be analysed thematically using the Framework method.

Ethics and dissemination

This study has been approved by the National Health Service Research Ethics Committee (Reference: 22/SC/0210). Written informed consent or verbal consent will be obtained prior to each interview. The findings from this study will be disseminated through peer- reviewed publications, conferences and lay summaries.

For peer review only - http://bmjopen.bmj.com/site/about/guidelines.xhtml

STRENGTHS AND LIMITATIONS OF THIS STUDY

- This study will use a UK population-based database comprising more than 30 million primary healthcare electronic records to facilitate a selection of a diverse sample of patients with multiple long-term conditions within the West Midlands in terms of key demographics and mix of co-occurring conditions.
- Including perspectives from both patients and HCPs will allow us to capture doubts, barriers or issues in using AI-based clinical support tools from the people that will be affected the most by the development and implementation of an effective AI tool.
- Use of a systematic methodology and extensive involvement of our patient and public advisory group have ensured our data collection tools are fit for purpose.
- Despite the strength of using a UK population-based database, the database may also provide limitations due to possibilities of under-representation of certain groups due to structural inequalities.
- Non-probability sampling and including patients only from the West Midlands may limit generalisability.

For peer review only

INTRODUCTION

One quarter of adults in England have two or more long-term health conditions.(1, 2) Having four or more long-term health conditions is strongly correlated with increasing age and has a significant contribution toward health service utilisation.(3) People with multiple long-term conditions are often prescribed multiple different medications (polypharmacy), which can lead to multiple side effects in addition to symptoms from their health conditions.(4) Evidence also suggests that polypharmacy can result in lower quality of life (5) and a three to four-fold increased risk in mortality among people aged 65 years or above.(6)

From the healthcare perspective, the more conditions a person has and more medications they take, the harder it is for healthcare professionals (HCPs) to consider all the factors when determining the best treatment plan, as clinical guidelines for one condition do not usually consider other existing health conditions.(7) Evidence is lacking on how to best treat health conditions for people with multiple long-term health conditions which in many cases, leads to excess treatment burden.(8) Due to limited resources for people with multiple long-term health conditions and the complex interaction between different disease conditions, primary care HCPs have identified the need for developing and adopting guidelines on how to best deliver and manage care for those with multiple health conditions.(9) Evidence based guidelines are typically established from findings of clinical trials for individual diseases; however, such trials often exclude people with multiple health conditions.(7) As a result, HCPs are provided with guidelines for individual conditions that are often too complex and heterogenous to combine or integrate to determine the best treatment option for individuals with multiple health conditions. Observational studies could be conducted using large scale population-based data (such as routinely collected electronic health records) to better understand the impact of medication on multiple health conditions; however, such studies are limited by the inability to account for individual patient medical histories, demographics (e.g. age, gender, ethnicity) and the variability in decisions made by HCPs providing care.

Artificial intelligence (AI) tools are one solution that is being suggested to overcome some of the limitations in guidelines and evidence base for patients with multimorbidity.(10) AI is broadly defined as the science of "machines [that] do things that would require intelligence if done by people".(11) Machine learning is a type of AI in which a computer with self-learning capacity can

For peer review only - http://bmjopen.bmj.com/site/about/guidelines.xhtml

generate predictive algorithms and identify patterns from data.(12) This has been successful at tackling complex problems outside of healthcare;(10) however, the capacity to process large amounts of information, systematically and reliably, faster than the human brain means there is considerable potential for AI in complex healthcare decision making. The Royal College of General Practitioners has recognised that there is vast potential for AI in general practice.(13) There are some working examples to date in other settings (e.g.: secondary care, intensive care) where AI-based tools have been developed and validated for risk stratification and patient outcome optimisation,(14) but application of AI-tools in primary care is still in its infancy.(15, 16)

The OPTIMising therapies, disease trajectories, and artificial intelligence (AI) assisted clinical management for patients Living with multiple long-term health conditions (OPTIMAL) study aims to produce an AI-based tool to be used in primary care settings for planning the best treatment strategies and predicting the next health condition that people with multiple health conditions might develop to inform screening, investigations, prevention and/or treatment. Using population-based anonymised data available from primary and secondary healthcare records, the OPTIMAL study will determine the trajectories of disease accumulations for people with multiple health conditions and the contribution of medicines on the trajectories. The team will then develop a predictive model for the next likely disease and the best treatment option in the context of multiple conditions and multiple treatment options and incorporate this into an AI-based decision-making tool that can be used by HCPs and patients for joint decision making for the best treatment plan.

For AI decision-making tools to be successfully implemented and effectively used in clinical practice, it is important that HCPs and patients trust, understand and see the value of using the tool. A 2021 systematic review of 23 qualitative studies exploring patient and public perspectives towards AI in the clinical setting found that the public were broadly positive about the concept of AI, but were concerned about the effectiveness of AI tools and felt that implementation should have human oversight.(17) However, in three-quarters of the included studies, discussions were based on AI as a hypothetical concept, rather than a real-world example (likely because few existed), which may limit the depth or specificity of the discussions.(17) Some qualitative studies have reported concerns from patients regarding the lack of a 'human touch' in using AI-technology, but most have optimism that AI-based tools could free up HCP time, leaving more time for patient interactions.(18) Studies of clinicians' attitudes to AI are also generally positive,

For peer review only - http://bmjopen.bmj.com/site/about/guidelines.xhtml

especially around the potential to take over mundane administrative tasks and synthesise data.(18-21) However, studies exploring UK GPs' perspectives, including a large survey of 720 respondents, found that GPs believe human empathy, communication and tailoring to individual patients' values could not be replicated by AI.(21-23) Another study examining GP perspectives on using an AI-based documentation tool found they had concerns about the impact on their professional autonomy and who would bear the medico-legal responsibility for any outcomes.(19) Those in managerial and regulatory roles (including clinicians) have also expressed concerns over regulatory oversight of AI, the transferability/generalisability of the tools and the challenge of adopting AI within existing systems.(9) This was felt to be especially difficult within primary care which involves multiple independent practices and deals with a high burden of what were thought of as 'non-digitisable' healthcare problems such as mental health and chronic illness.(9)

Most existing studies exploring perspectives on AI have treated it as a broad and hypothetical concept.(17, 18) Furthermore, limited evidence that describes patient perceptions of using AI-based tools for managing existing conditions or multiple health conditions.(21) Exploration of current and informed perspectives using a real-world example of an AI-based tool is needed. Insights from HCPs regarding the challenge of implementing this technology in the management of multiple coexisting health conditions in primary care will be especially valuable.

Input from patients with multiple health conditions and HCPs on using the AI-based tool is critical in its development and implementation to ensure any specific barriers for the use of the tool can be appropriately addressed. Therefore, another component of the OPTIMAL study is a qualitative study which this protocol describes.

Theoretical framework

Theoretical frameworks can be used at all stages in qualitative research to allow for a deeper understanding of how people and the cultures and organisations they are part of operate, interact and behave.(24) Several theoretical frameworks have been used to conceptualise attitudes and barriers to the introduction of AI in healthcare settings.(9, 25, 26) To explore the perspectives of general practitioners (GPs) on AI in primary care in Germany, Buck et al.(25) built on Rosenberg and Hovland's (27) previous work on the construct of attitudes to change (Figure 1). This model distinguishes the affective (emotional reactions, empathy, and feelings), cognitive (ideas and knowledge), and behavioural (extent to which attitudes predict actions and intentions to act)

For peer review only
For peer review only - http://bmjopen.bmj.com/site/about/guidelines.xhtml

dimensions to attitudes to use of AI-enabled systems in healthcare.(27, 28) Individual characteristics such as age and prior experience with AI, and environmental influences such as the positive attitude from primary care professional bodies also influenced attitudes to AI. Given that the application of AI in general practice is largely hypothetical at present, current attitudes to AI from potential users are critical to the development of technologies that could then be implemented in practice. Therefore, we did not feel it appropriate to overly constrain the work by introducing theory, and therefore a particular slant, from the very outset. However, we will apply Buck et al.'s theoretical lens at the analysis stage. This lens will be used as a framework to further explore the data from HCP interviews to provide a broader conceptual understanding of how the affective, cognitive and behavioural components of attitude determine how HCPs evaluate their views on AI in managing multiple health conditions.

Study aim

Through semi-structured interviews (SSIs), this qualitative study aims to understand patients' and HCPs' perceptions of the advantages and disadvantages presented by AI-informed decision-making compared to clinician directed decision-making for the management of multiple health conditions.

METHODS AND ANALYSIS

Study design and participants

This qualitative study will be conducted using SSIs (29) with two key groups of stakeholders. The first group will include people aged 18 years or older, with more than one long-term health condition who are registered with a GP in the West Midlands County in England that contributes to the Clinical Practice Research Datalink (CPRD) Aurum database. Sixty-three conditions were considered when assessing eligibility (Supplementary material); these were derived through existing evidence (30) and discussions with the OPTIMAL Patient and Public Advisory Group (PPAG). Patients with a terminal diagnosis (prognosis of less than 12 months) will be excluded to avoid undue distress as well as those deemed by their GP not to have the capacity to consent or participate. For the second group, HCPs currently working in the UK involved in managing patients with multiple health conditions will be eligible to take part. A diverse range of

For peer review only - http://bmjopen.bmj.com/site/about/guidelines.xhtml

professionals including but not limited to geriatricians, GPs, community pharmacists and specialist nurses will be eligible and invited to participate.

Recruitment

CPRD Aurum is a population-based database of electronic primary healthcare records of anonymised data on diagnoses, tests, prescriptions and demographics,(31) and will be used in the first instance to select eligible patients (Figure 2). Prevalence and incidence data of many conditions in CPRD Aurum have been validated and demographics have been deemed representative of the general population in England.(31) Anonymised patient identifiers of patients from practices within the West Midlands will be accessed using the Data Extraction for Epidemiological Research (DExtER) software.(32) The eligible list of patients' unique CPRD identification numbers will be uploaded to CPRD's interventional research service platform (IRSP). CPRD will contact GP practices asking them to take part in the study. Initially 300 eligible patients from 10 GP practices will be selected and invited to participate. Each practice who takes part will be provided a list of eligible patients together with their CPRD ID number through the IRSP dashboard within their practice. Once this list has been received by the practices, either the practice manager, nurse or GP will view the eligible patients' details. They will review their medical history to ensure they meet the eligibility criteria. Once eligibility is confirmed, these patients will be sent an invitation letter and participant information sheet (Supplementary material) from their GP. Interested patients will be asked to call or email the OPTIMAL research team, at which point they can ask any questions about the study.

To augment this recruitment method for patients and ensure we get a diverse sample, we will also circulate recruitment flyers through voluntary, third sector and patient support and community groups, including but not limited to Citizens UK (33) and Birmingham Voluntary Services Council,(34) and through help with from the OPTIMAL PPAG. Flyers will include information on eligibility and how to contact the OPTIMAL team to learn more about the study, ask questions, and receive a copy of the participant information sheet before deciding if they would like to take part. The OPTIMAL team will select up to 30 eligible and interested patients for participation (or until data saturation is agreed by the researchers).(35, 36) Saturation will be agreed when no additional themes emerge from the data.(36) Purposive sampling (37) will be used to ensure maximal diversity in the characteristics of the participants in terms of age, gender, ethnicity, socio-economic status and number and types of long-term health conditions.

For peer review only - http://bmjopen.bmj.com/site/about/guidelines.xhtml

HCPs will be recruited through known contacts and British healthcare professional bodies. This will include the British Geriatric Society, Royal College of General Practitioners, Society for Academic Primary Care, Royal College of Physicians, National Pharmacy Association, and the Royal College of Nursing. We will contact these organisations and request they send a letter or email describing the study to all eligible HCPs (Supplementary material). Any HCP interested in taking part will be instructed to email or phone the OPTIMAL research team directly. We aim to recruit until data saturation (36) is reached, up to a maximum of 30 HCPs using purposive sampling based on profession, age, years of experience, sex, ethnicity and place of practice (urban/rural, teaching/non-teaching etc).

Study materials

To encourage a rich dialogue during these interviews, a case vignette (38) (Supplementary material) will be used in conjunction with a semi-structured topic guide (one for patients and one for HCPs; Supplementary material). The topic guides will explore patients' and HCPs' attitudes towards AI-informed clinical decision-making compared to clinician directed decision-making, the perceived advantages and disadvantages of both methods and attitudes to risk management. These topic guides were developed based on the literature and guidance from the OPTIMAL PPAG.

The case vignettes were co-developed by clinicians within the OPTIMAL team and the PPAG to reflect an expected decision-making process for a patient presenting with a common combination of multiple health conditions with both physical and mental comorbidities. HCPs will be asked how their decision-making process compares to potential outputs of the AI-based tool whereas patients will be asked if the vignette's trajectories fit with their owned lived experiences in terms of the process for receiving their diagnoses and prescriptions. Participants will be encouraged to elaborate on their answers using prompts, probes and follow up questions. The topic guide questions and case vignette were piloted with two patients and one HCP. Following these pilot interviews, the length of the case vignettes was shortened as it was said to be too long during the pilot; no other changes were made.

Data collection

The SSIs will be conducted virtually via Zoom or Teams, by telephone or face-to-face (39, 40) according to participant preference. There will be two interviewers; one will conduct the interview with patients and one will interview HCPs. All interviews will be audio recorded. Age, sex, ethnicity and number of existing conditions will be collected from the patients. Age, sex, ethnicity, profession,

For peer review only - http://bmjopen.bmj.com/site/about/guidelines.xhtml

and years of experience will be collected from the HCPs who take part. Prior to the interview, all participants will be provided with a summary explanation of AI (Supplementary material) in general and healthcare contexts.

Data analysis

Interviews will be transcribed verbatim. NVivo (version 10 for Windows) will be used for data management. The de-identified transcripts generated from the recordings will be passed through word protectors and will be stored with access granted only for the data analysing team, allowing broad perspectives on the data. An inductive thematical analysis will initially be conducted on both HCP and patient transcripts whereby codes will be assigned line-by-line using the Framework Method.(41) Interviews will be analysed concurrently and iteratively to inform a reflexive process and create a cycle of data collection, analysis and planning what questions to add or amend in subsequent interviews. Similar codes will be combined and themes will be identified in an analytical framework. Ten percent of HCP and patient transcripts will be coded by a second researcher who did not facilitate the interviews; findings will be compared, and any disparities will be resolved through discussion with the wider team. After reviewing and revising, the final themes will be determined and the interpretations explored. A summary of overall themes will be sent to patient and HCP participants for comment.

A further analysis of HCP transcripts will be carried out using Buck et al.'s theoretical framework as a starting point for a deductive analysis to explore and separate out participants' affective, cognitive and behavioural components of their attitudes toward AI use in managing multiple health conditions. Codes will be assigned to Buck et al.'s framework to see if and in what ways the data fits the model and deepens understanding of attitudes toward AI. This framework has not previously been applied to patient perspectives; thus, we do not intend to apply it a priori to the patient transcripts.

As the data coding framework and overall themes emerge these will be discussed with the PPAG and members of the multidisciplinary research team to establish any further areas for exploration or clarification in subsequent interviews. A list of overall and individual themes for patients, and HCPs will be compiled to allow for cross group/individual and purposive sampling characteristics comparison and to understand how individual characteristics of HCPs (e.g. age and prior

For peer review only

For peer review only - http://bmjopen.bmj.com/site/about/guidelines.xhtml

experience with AI) and environmental influences (e.g. current working environment, media influences, available technology in the workplace) may influence perspectives.

Patient and public involvement

Members of the public and patients with experience of multiple health conditions have been involved in every stage of the OPTIMAL study. A PPAG was set up at the inception stage of the study comprising eight people who have lived experience of multiple health conditions (either directly or as a carer). We ensured that equality, diversity and inclusivity were prioritised and people of varied age, ethnicity, sex, geographic location in the UK and experience with multiple health conditions were invited to take part. Our PPAG contributed to the development of the study including objective setting, design and study materials through regular meetings. The topic guide, participant information document, consent form and invitation letter for patients were all reviewed by and improved according to feedback from our PPAG before ethical approval was sought. Throughout the study, meetings are expected to take place every three months with the PPAG. It is anticipated that these meetings will be particularly useful during the recruitment, data collection and analyses stages of the qualitative component this protocol describes to ensure the study aims and objectives are being met. To date, the impacts of the PPAG have included: expanding the range of HCPs invited to take part (i.e. to include pharmacists), improved the inclusiveness of the recruitment strategy and amending the design of the case vignettes to be more representative of their experiences.

ETHICS AND DISSEMINATION

Ethical approval for the study was obtained from National Health Service (NHS) Research Ethics Committees (REC) (Reference: 22/SC/0210). Ethical approval to use the anonymised data from the CPRD dataset to select the eligible participants was obtained from the CPRD Expert Review Committee and the Central Advisory Committee (Reference:21_000683).

GPs will not know who decides to participate from the eligible patients they contact for recruitment and likewise, none of the healthcare professional bodies will know which members decide to participate. Written informed consent will be obtained from the face-to face interviews and either electronically completed written consent forms or audio informed consent will be collected from the video or phone interviews; in the latter scenario, researchers will produce a written version of the consent on their behalf. If a participant withdraws from the study within two weeks of the interview,

For peer review only - http://bmjopen.bmj.com/site/about/guidelines.xhtml

data collected from the participant will be securely destroyed. Otherwise, all data provided from consented participants will be used in the final analysis.

Data collection began with HCPs in October 2022 and are expected to be completed by September 2023; data collection with patients is expected to begin in August 2023 and completed by November 2023; analyses will be completed by February 2024. The final study report will be circulated to the relevant stakeholders and the summary of the final report will be available to the public by the National Institute for Health and Care Research (NIHR) and accompanied by a plain language summary. Furthermore, the study findings will be shared through peer-reviewed publications, public engagement activities and national or international conferences.

DISCUSSION

Programming an effective and accurate AI-based decision-making tool using advanced machine learning and bioinformatics algorithms is one way to improve our understanding of how to reduce complications and additional comorbidities and safely manage polypharmacy in patients with multiple health conditions. However, patients and HCPs may feel there are barriers, risks and challenges for the use of AI in healthcare as well as benefits. Understanding these perceived barriers and potential risks is vital for effective implementation. This qualitative study will capture patients and HCPs perspectives on an AI-informed decision-making tool developed as part of the OPTIMAL study.

A large proportion of people aged 65 years or older have multiple health conditions.(42) AI-based tools have great potential in optimising disease management in the context of multiple health conditions and polypharmacy where the use of clinical trials are greatly limited. A major strength of this study is the use of data from the CPRD Aurum database to select eligible patients. CPRD Aurum is representative of the UK general population in terms of geographic spread, age, gender, deprivation and patterns of diseases.(43) This will facilitate the selection of a diverse sample of patients with multiple health conditions in terms of known population demographics. Another strength is the study's dedicated PPAG; they have and will continue to play an important role at every stage of the study ensuring that this research and the AI-based tool are of value to patients and carers with multiple health conditions. This also provides a unique opportunity for open dialogue between patients and members of the public, researchers and clinicians so they understand each other's perspectives and can discuss distinct challenges and learn from each other. By conducting qualitative interviews, this

For peer review only - http://bmjopen.bmj.com/site/about/guidelines.xhtml

study will provide an in-depth understanding of patient and HCP perspectives which is vital for optimising the development and future implementation of an effective AI tool.(18) The systematic methodology of the Framework Method, input from the PPAG and respondent validation will increase the trustworthiness of the conclusions while reducing researcher bias.(44) A limitation of the study to note is the non-probability sampling and involving patients only from the West Midlands which may limit the generalisability of the findings to patients from other regions in England, however the high socio-demographic diversity (45) of the area will aid understandings about how perceptions may vary between different demographic groups.

To conclude, our study will elucidate the perceptions of HCPs caring for patients with multiple health conditions and patients living with multiple health conditions on the use of AI-based clinical decision support for the management of multiple health conditions. AI-based tools have the potential to improve the care of multiple health conditions through better prescription management and de-prescription; thus, reduce polypharmacy, side-effects, drug burden and ultimately excess morbidity and premature mortality. It is therefore vital for patients and HCPs to raise their concerns and potential barriers regarding the use of an AI-based tool for the tool to effectively work during the shared decision-making process. Our findings will enable a better understanding on how an AI-based tool can be developed and implemented in way that is acceptable to and effectively developed and implemented in a way that is trustworthy and allows effective use by HCPs and is acceptable and accessible to the patient groups who are likely to benefit from the tool the most.

Acknowledgements

The authors would like to thank the members of the PPAG (currently comprised of: Robert Jasper, Christine Michael, Jenny Negus, Gillian Richards, Lynne Wright and Janice Connelly) for their invaluable contribution to the conceptualisation and development of this study thus far.

Contributors

In collaboration with the PPAG, NJG, TEG, JC, SF, TM, SH, ADE, FC, TAJ, KN and SG contributed to the conceptualisation of the study. JC and SF drafted the study tools, led the ethics

For peer review only - http://bmjopen.bmj.com/site/about/guidelines.xhtml

application and piloted the topic guides and case vignettes with supervision from FC, TAJ, KN and SG. NJG and TEG drafted all versions of the manuscript with supervision from FC. TAJ, KN and SG are senior authors overseeing the project.

Funding

This work is independent research funded by the National Institute for Health and Care Research (NIHR) (OPTIMising therapies, disease trajectories, and AI assisted clinical management for patients Living with complex multimorbidity (OPTIMAL study), NIHR202632). The views expressed in this publication are those of the author(s) and not necessarily those of NIHR or The Department of Health and Social Care.

Competing interests

The author(s) declared no potential conflicts of interest with respect to the research, authorship, and/or publication of this article. The researchers involved in recruitment, data collection and data analysis for the qualitative study are not the same researchers who will be developing the AI-based tool.

For peer review only

References

1. Skou ST, Mair FS, Fortin M, Guthrie B, Nunes BP, Miranda JJ, et al. Multimorbidity. Nature Reviews Disease Primers. 2022;8(1):48.
2. Cassell A, Edwards D, Harshfield A, Rhodes K, Brimicombe J, Payne R, et al. The epidemiology of multimorbidity in primary care: a retrospective cohort study. British Journal of General Practice. 2018;68(669):e245-e51.
3. Johnston MC, Crilly M, Black C, Prescott GJ, Mercer SW. Defining and measuring multimorbidity: a systematic review of systematic reviews. Eur J Public Health. 2019;29(1):182-9.
4. Aoki T, Yamamoto Y, Ikenoue T, Onishi Y, Fukuhara S. Multimorbidity patterns in relation to polypharmacy and dosage frequency: a nationwide, cross-sectional study in a Japanese population. Scientific Reports. 2018;8(1):3806.
5. Schenker Y, Park SY, Jeong K, Pruskowski J, Kavalieratos D, Resick J, et al. Associations Between Polypharmacy, Symptom Burden, and Quality of Life in Patients with Advanced, Life-Limiting Illness. Journal of General Internal Medicine. 2019;34(4):559-66.
6. Jørring Pallesen AV, Kristiansen M, Westendorp RGJ, Mortensen LH. Polypharmacy occurrence and the related risk of premature death among older adults in Denmark: A nationwide register-based cohort study. PLoS One. 2022;17(2):e0264332.
7. Hughes LD, McMurdo ME, Guthrie B. Guidelines for people not for diseases: the challenges of applying UK clinical guidelines to people with multimorbidity. Age Ageing. 2013;42(1):62-9.
8. Bower P, Macdonald W, Harkness E, Gask L, Kendrick T, Valderas JM, et al. Multimorbidity, service organization and clinical decision making in primary care: a qualitative study. Fam Pract. 2011;28(5):579-87.
9. Morrison K. Artificial intelligence and the NHS: a qualitative exploration of the factors influencing adoption. Future Healthc J. 2021;8(3):e648-e54.
10. Raghupathi W, Raghupathi V. Big data analytics in healthcare: promise and potential. Health Inf Sci Syst. 2014;2:1-10.
11. Minsky ML. Semantic information processing: The MIT Press; 2003.
12. Panch T, Szolovits P, Atun R. Artificial intelligence, machine learning and health systems. J Glob Health. 2018;8(2).
13. Royal College of General Practitioners. Artificial Intelligence and Primary Care. Royal College of General Practitioners; 2019.
14. Giordano C, Brennan M, Mohamed B, Rashidi P, Modave F, Tighe P. Accessing artificial intelligence for clinical decision-making. Front Digit Health. 2021;3:645232.
15. Haug CJ, Drazen JM. Artificial intelligence and machine learning in clinical medicine, 2023. N Engl J Med. 2023;388(13):1201-8.
16. Karwath A, Bunting KV, Gill SK, Tica O, Pendleton S, Aziz F, et al. Redefining β-blocker response in heart failure patients with sinus rhythm and atrial fibrillation: a machine learning cluster analysis. Lancet. 2021;398(10309):1427-35.
17. Young AT, Amara D, Bhattacharya A, Wei ML. Patient and general public attitudes towards clinical artificial intelligence: a mixed methods systematic review. Lancet Digit Health. 2021;3(9):e599-e611.
18. Hogg HDJ, Al-Zubaidy M, Talks J, Denniston AK, Kelly CJ, Malawana J, et al. Stakeholder Perspectives of Clinical Artificial Intelligence Implementation: Systematic Review of Qualitative Evidence. J Med Internet Res. 2023;25:e39742.
19. Kocaballi AB, Ijaz K, Laranjo L, Quiroz JC, Rezazadegan D, Tong HL, et al. Envisioning an artificial intelligence documentation assistant for future primary care consultations: A co-design study with general practitioners. J Am Med Inform Assoc. 2020;27(11):1695-704.

For peer review only - http://bmjopen.bmj.com/site/about/guidelines.xhtml

20. Martinho A, Kroesen M, Chorus C. A healthy debate: Exploring the views of medical doctors on the ethics of artificial intelligence. Artif Intell Med. 2021;121:102190.
21. Scott IA, Carter SM, Coiera E. Exploring stakeholder attitudes towards AI in clinical practice. BMJ Health Care Inform. 2021;28(1).
22. Blease C, Kaptchuk TJ, Bernstein MH, Mandl KD, Halamka JD, DesRoches CM. Artificial intelligence and the future of primary care: exploratory qualitative study of UK general practitioners' views. J Med Internet Res. 2019;21(3):e12802.
23. Blease C, Bernstein MH, Gaab J, Kaptchuk TJ, Kossowsky J, Mandl KD, et al. Computerization and the future of primary care: a survey of general practitioners in the UK. PloS One. 2018;13(12):e0207418.
24. Reeves S, Albert M, Kuper A, Hodges BD. Why use theories in qualitative research? BMJ. 2008;337.
25. Buck C, Doctor E, Hennrich J, Jöhnk J, Eymann T. General Practitioners' Attitudes Toward Artificial Intelligence-Enabled Systems: Interview Study. J Med Internet Res. 2022;24(1):e28916.
26. Darcel K, Upshaw T, Craig-Neil A, Macklin J, Steele Gray C, Chan TCY, et al. Implementing artificial intelligence in Canadian primary care: Barriers and strategies identified through a national deliberative dialogue. PLOS ONE. 2023;18(2):e0281733.
27. Rosenberg MJ. Attitude Organization and Change; an Analysis of Consistency Among Attitude Components. 1960.
28. Chang A, editor Common Misconceptions and Future Directions for AI in Medicine: A Physician-Data Scientist Perspective. Artificial Intelligence in Medicine; 2019 2019//; Cham: Springer International Publishing.
29. Kallio H, Pietilä AM, Johnson M, Kangasniemi M. Systematic methodological review: developing a framework for a qualitative semi-structured interview guide. J Adv Nurs. 2016;72(12):2954-65.
30. Ho IS, Azcoaga-Lorenzo A, Akbari A, Davies J, Khunti K, Kadam UT, et al. Measuring multimorbidity in research: Delphi consensus study. BMJ Med. 2022;1(1).
31. Herrett E, Gallagher AM, Bhaskaran K, Forbes H, Mathur R, van Staa T, et al. Data Resource Profile: Clinical Practice Research Datalink (CPRD). Int J Epidemiol. 2015;44(3):827-36.
32. Gokhale KM, Chandan JS, Toulis K, Gkoutos G, Tino P, Nirantharakumar K. Data extraction for epidemiological research (DExtER): a novel tool for automated clinical epidemiology studies. Eur J Epidemiol. 2021;36(2):165-78.
33. Citizens UK. Bringing communities together to win change [Available from: https://www.citizensuk.org/.
34. Birmingham Voluntary Service Council. BVSC the centre for voluntary action [Available from: https://www.bvsc.org/.
35. Patton MQ. Enhancing the quality and credibility of qualitative analysis. Health Serv Res. 1999;34(5 Pt 2):1189-208.
36. Morse JM. Determining Sample Size. Qualitative Health Research. 2000;10(1):3-5.
37. Palinkas LA, Horwitz SM, Green CA, Wisdom JP, Duan N, Hoagwood K. Purposeful sampling for qualitative data collection and analysis in mixed method implementation research. Adm Policy Ment Health. 2015;42:533-44.
38. Tremblay D, Turcotte A, Touati N, Poder TG, Kilpatrick K, Bilodeau K, et al. Development and use of research vignettes to collect qualitative data from healthcare professionals: A scoping review. BMJ Open. 2022;12(1):e057095.
39. Keen S, Lomeli-Rodriguez M, Joffe H. From challenge to opportunity: virtual qualitative research during COVID-19 and beyond. International journal of qualitative methods. 2022;21:16094069221105075.
40. Sturges JE, Hanrahan KJ. Comparing telephone and face-to-face qualitative interviewing: a research note. Qual Res. 2004;4(1):107-18.

For peer review only - http://bmjopen.bmj.com/site/about/guidelines.xhtml

41. Gale NK, Heath G, Cameron E, Rashid S, Redwood S. Using the framework method for the analysis of qualitative data in multi-disciplinary health research. BMC Med Res Methodol. 2013;13:117.
42. Barnett K, Mercer SW, Norbury M, Watt G, Wyke S, Guthrie B. Epidemiology of multimorbidity and implications for health care, research, and medical education: a cross-sectional study. Lancet. 2012;380(9836):37-43.
43. Wolf A, Dedman D, Campbell J, Booth H, Lunn D, Chapman J, et al. Data resource profile: Clinical Practice Research Datalink (CPRD) Aurum. Int J Epidemiol. 2019;48(6):1740-g.
44. Birt L, Scott S, Cavers D, Campbell C, Walter F. Member Checking: A Tool to Enhance Trustworthiness or Merely a Nod to Validation? Qual Health Res. 2016;26(13):1802-11.
45. Patel K NA. Diversity and cultural leadership in the West Midlands. A report for the West Midlands leadership commission. 2018 March.

Figure 1. Buck et al.'s model of the general practitioners' determinants of attitudes toward artificial intelligence (AI)–enabled systems

This figure is reproduced under the terms of the Creative Commons Attribution License (https://creativecommons.org/licenses/by/4.0/) from work published in the Journal of Medical Internet Research,(25) available at https://www.jmir.org/2022/1/e28916.

Figure 2. Flowchart of study participant recruitment

CPRD: Clinical Practice Research Datalink; IRSP: interventional research service platform; OPTIMAL: GP: general practitioners; OPTIMising therapies, disease trajectories, and artificial intelligence (AI) assisted clinical management for patients Living with multiple long-term health conditions.

For peer review only

For peer review only - http://bmjopen.bmj.com/site/about/guidelines.xhtml

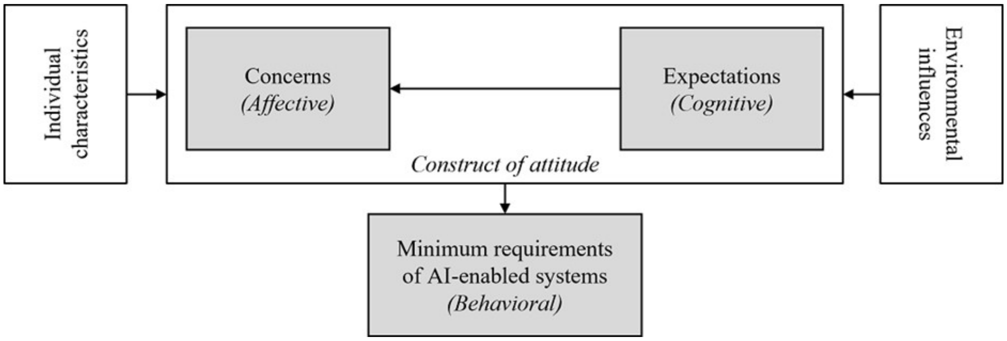

Figure 1. Buck et al.'s model of the general practitioners' determinants of attitudes toward artificial intelligence (AI)–enabled systems.

165x55mm (144 x 144 DPI)

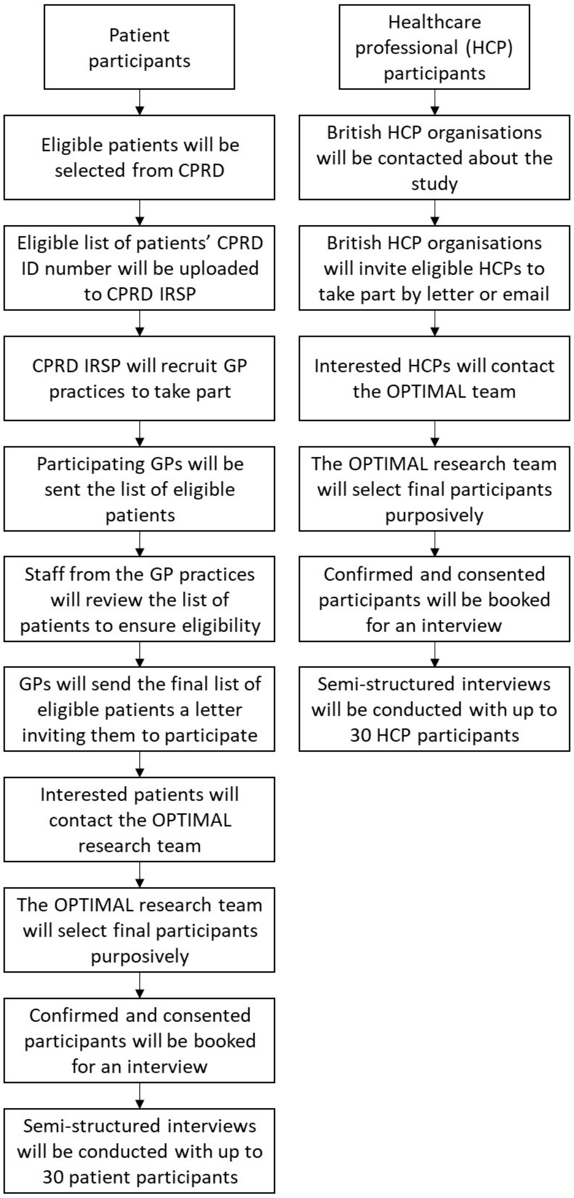

Flow chart of study participant recruitment

132x277mm (150 x 150 DPI)

Supplementary Material 1

List of 63 conditions considered when determining eligible patients with 2 or more conditions.

Heart failure
Atrial fibrillation (AF)
Stroke
Hypertension
Ishaemic heart disease/ coronary artery disease (IHD)
Peripheral vascular disease (PVD or PAD)
Heart valve disorders
Aortic aneurysm
Type 1 diabetes
Type 2 diabetes
Chronic kidney disease (CKD)
Depression
Anxiety
Bipolar disorder
Eating disorder
Schizophrenia
Post traumatic stress disorder (PTSD)
Autism
Drug/alcohol misuse
Alcoholic liver disease
Non-alcoholic fatty liver disease (NAFLD)
Other (or non-specified) chronic liver disease
Inflammatory bowel disease (IBD)
Irritable bowel syndrome (IBS)
Dementia
Parkinson's disease
Epilepsy
Cancer (excluding BCC)
Haematological cancer (leukaemia/lymphoma/ myeloma)
Asthma
Chronic obstructive pulmonary disease (COPD)
Obstructive sleep apnoea (OSA)

For peer review only

Bronchiectasis and cystic fibrosis
Eczema
Allergic rhinitis (hayfever)
HIV/AIDS
Osteoporosis
Osteoarthritis
Rheumatoid arthritis
Gout
Systemic lupus erythematosus (SLE)
Sjogren's disease
Systemic sclerosis
Polymyalgia rheumatica / Giant cell arteritis (PMR/GCA)
Fibromyalgia/ chronic fatigue (CFS)
Polycystic ovarian syndrome (PCOS)
Endometriosis
Hypothyroidism
Hyperthyroidism
Addison's disease
Multiple sclerosis
Vision impairment long term
Hearing impairment long term
Meniere's disease
Peripheral neuropathy
Intellectual disabilites
Down syndrome
Pernicious anaemia
Sickle cell anaemia
Psoriasis
Psoriatic arthritis
Interstitial lung disease
Haemochromatosis

For peer review only

Supplementary Material 2

Information sheet/ Information letter

Practice address/contact details

<<Patient Address>>

<<Date>>

OPTIMIsing therapies, disease trajectories, and AI assisted clinical management for patients Living with complex multimorbidity (OPTIMAL study)

Your Unique Reference Code is: CPRD *<<Global ID>>*

Dear *<<Patient name>>*

We would like to invite you take part in a research study. The research team are hoping to speak to people who have multiple (four or more) long-term health conditions to discuss their thoughts about how artificial intelligence (AI) may help them and their health care professionals manage these conditions. They will also be speaking to health care professionals to help understand their views.

Before you decide whether you would like to take part in an interview, please read the enclosed participant information sheet that explains the study in more detail and what would happen if you agree to take part.

If you would like to participate in an interview, please contact a member of the research team whose details are given below.

The research team:
OPTIMAL Team
Institute of Allied Health Research, University of Birmingham, B15 2TT

For more information, contact:
[name, email and contact number of study lead]

Thank you for taking the time to consider taking part in the study.

Yours sincerely,

<<GP sign off>>

For peer review only

UNIVERSITY OF BIRMINGHAM

NHS National Institute for Health Research

OPTIMIsing therapies, disease trajectories, and AI assisted clinical management for patients Living with complex multimorbidity (OPTIMAL)

For peer review only

Participant information sheet: patient interviews

Introduction

We would like to invite you to take part in a research study. Before you decide to take part, we would like to explain why we are doing this study and what your involvement will mean for you. Please take time to read this information sheet and discuss it with others if you wish. Ask us if there is anything that is not clear or if you would like more information before deciding whether or not to take part. Our contact details are at the end of this information sheet.

What is the study about?

The study aims to use artificial intelligence (AI) to produce computer programmes and tools that will help improve the treatment and choice of medications in patients with clusters of multiple long-term conditions (we say these people have complex multimorbidity or cMM). Artificial intelligence (or AI) is a computer system that can conduct tasks that would normally need human intelligence. There are examples of AI being used in our everyday lives, with applications and software such as Spotify, Amazon and BBC iplayer. These 'apps' and on-line websites function by predicting what kind of music, TV programmes, or general purchases we may like or want. They do this by using a computer programme that can observe what categories and kinds of TV, Music etc. we watched or bought in the past and use this data to predict what we are a likely to choose in the future.

The machine learning branch of AI is also used in things like self-driving cars, language translations and it is being developed for use in healthcare. Newer AI methods makes it easier to process large amount of health data in a short time. These AI methods can give doctors and patients information that may help improve the care of people with multiple long-term health conditions

AI can be used in healthcare to help guide the diagnosis of long-term health conditions, plan the best treatment strategies, and predict the next health condition that people might develop. This is especially relevant for people who have several different health conditions

because the guidelines that doctors and healthcare professionals may use for one condition does not routinely consider other health conditions.

AI-based decision-making tools (computer programs) for managing multiple health conditions may help patients and healthcare professionals make more well-informed shared decisions. Patients and healthcare professionals may see many benefits to the use of AI in healthcare. However, they may be concerned this could be harmful or that it could affect the relationships between healthcare professionals and patients. We want to speak to patients and healthcare professionals to understand their views about AI in healthcare. It is important in healthcare research that we reflect these experiences in any future plans for the use of AI in a healthcare setting.
Data collected from this study will give new insights to how healthcare professionals and people with multiple long-term health conditions view AI-based decision-making tools. It will also tell us which factors in the computer program are important to them and what options they prefer. This information will also be used to help to develop how these tools are used in clinical practice.

How will we do this?

1. By using artificial intelligence (AI) methods with electronic health records to generate data models that tell us how the different mixes of conditions arise over time and how certain drugs can make this better or worse.

2. By asking people with cMM and doctors about their knowledge and views about using AI to make decisions about health care.

3. By using AI computer techniques to combine data, and together with the input from people with cMM and doctors, develop a computer program. This will predict which drug we should give and when we should give it to someone with cMM to reduce risk and bring about maximum benefit. It will also tell us what disease people may get next. These predictions will be based upon data gathered from a large number of patients who have similar conditions and prescribed medication.

4. By examining the best way to present information in the AI tool to people with cMM and doctors by asking them about what is important to them and what options they prefer.

Our team includes patients, public members, and world leading experts from universities with expertise in biology, AI, medicine, health service research, public health, and general practice. Working as a multidisciplinary team we hope to improve the health and care of people with cMM.

Why have I been asked?

You have been invited because we would like to talk to you about your experiences living with four or more long-term health conditions.

Do I have to take part?

For peer review only

Taking part in this study is completely optional, the decision about whether to take part is up to you. Whether you decide to take part or not will have no effect upon your healthcare and treatment and you can leave the study at any time, without giving a reason.

What will happen to me if I take part?

If you choose to take part, you can contact the research team by telephone or email (details below). The researcher can provide more information about the study, and answer any questions you may have.
Please note that because we would like to interview a range of people depending upon certain characteristics, we will ask you for some basic details including your age, ethnicity, gender and number of health conditions). It may take a number of weeks before we can to arrange an interview date. In the event that we do not require your assistance, we will contact you to let you know.
The interviews will take place either face-to-face or remotely using videoconferencing or telephone at a time convenient to you. The interview should last for around 60 minutes, although you can ask for a break or stop the interview at any time.

The interviewer will ask questions about your thoughts on how AI informed decision making (the use of computer programmes) compares with doctors making decisions about your health.

We will also show you an example of a patient with several long-term conditions and how their health care has been managed using a computer programme, for example, how the computer programme has helped decide what medications they may need. We will ask you what kinds of things are important to know if a computer is informing these kinds of decisions about the medications you are prescribed your health in general.

If you agree to take part, we would like to audio record the interview using a secure recording device. After the interview, the recording will be written down (in a transcript) but all names and place names will be removed and no comments will be linked to you or any other person. The recording and transcript will be kept completely confidential using a study ID code and only the University evaluation team will have access to them.

At the end of the interview, you will be asked if you would be willing to be contacted at a later date to consider involvement in the second stage of the study. This stage will aim to explore preferences in terms of how computer programmes for AI directed clinical decision making are presented. For example, how the computer displays information, and what things patient think are important to see.

What are the possible benefits of taking part?

Although there is no direct benefit to you if you take part in an interview, the interviews will give us important information that should help patients with cMM along with the doctors who manage their conditions.

For peer review only

Will I receive any financial reimbursement for taking part?

You will be offered a £15 voucher (Amazon or Love 2 Shop) as a thank you for taking part in the interview.

What are the possible risks and disadvantages of taking part?

It may be upsetting to talk about living with multiple long-term conditions and your health care experiences. If this happens, we can stop or pause the interview at any time. We have put together a list of organisations that you can get in contact with who may be able to help. Many people have said that they find it helpful to talk to researchers about their experiences of living with conditions.
Taking part in the interview will take some of your time. However, you can choose a time and date that is suitable for you.
In light of COVID-19, we will adhere to current government guidelines and local procedures in order to minimise the risk of exposure. Support for participants will be provided to set up an online conferencing link, or by telephone according to participant preference. Researchers will have evidence of vaccination and/or negative results from a lateral flow test prior to interview if face-to-face.

What if there is a problem?

If you have any concerns, please speak to a member of the research team in the first instance, you can get in touch with us using the contact details at the end of this information sheet. We will do our best to answer your questions. If your concerns are not addressed and you wish to make a formal complaint, you can refer to the Patient Liaison Services (PALS) 0121 424 0808 PALS@uhb.nhs.uk.

Will my taking part in the study be kept confidential?

All information collected about you for this study will be subject to the General Data Protection Regulation and Data Protection Act 2018 for health and social care research and will be kept strictly confidential.

All audio-recordings will be kept for 10 years after the end of the study and then destroyed. Documentation and data from this part of the study will be securely stored at the University of Birmingham for 10 years.

How will we use information about you?

The University of Birmingham is the Sponsor for this study and this means that the University of Birmingham are responsible for looking after your information and using it properly. University of Birmingham and the NHS will keep identifiable information about you for at least 10 years after the study has finished, to allow the results of the study to be verified if needed. Information collected from you will include:

- Name
- Age

For peer review only

- Ethnicity
- Gender
- Number of long-term conditions

People who do not need to know who you are will not be able to see your name or contact details. Your data will have a unique study number instead and all information will be kept safe and secure. In the research team, you will be identified using your unique study number.

All information collected by the Sponsor, including a copy of your signed consent form, will be securely stored at the research study office at the University of Birmingham on paper and electronically and will only be accessible by authorised personnel. The only people in the University of Birmingham who will have access to information that identifies you will be people who manage the study or audit the data collection process.

The audio recordings from the interview will be transcribed by a transcription company which has been approved for transcription of medical data. If you agree to take part in the interview study, your name will not be on the recording and we will remove your name from the interview transcripts to keep your identity confidential. Direct quotes may be used in publications but these will be numbered and anything that could identify you will be removed. Nothing that you say will be fed back to the doctors and nurses involved in your care as coming from you.

What are your choices about how your information is used?

You can choose to stop taking part in the study at any time, without giving a reason, but we will keep information about you that we already have. If you agree to take part in this study, you will have the option to take part in future research using your data saved from this study. To safeguard your rights, we will use the minimum personally identifiable information possible. You can find out more about how your information will be used at https://www.birmingham.ac.uk/privacy/index.aspx.

The NHS and the University of Birmingham will use your name and contact details to contact you about the research study, and make sure that relevant information about the study is recorded and to oversee the quality of the study. Individuals from the University of Birmingham and regulatory organisations may look at your research records to check the accuracy of the research study.

All individuals who have access to your information have a duty of confidentiality to you. Under the provisions of the General Data Protection Regulation (GDPR) 2018, you have the right to know what information the Trial Office has recorded about you. If you wish to view this information, or find more about how we use this information, please contact the University of Birmingham's Data Protection Officer at the address below.

Where can you find out more about how your information is used?

For peer review only

If you would like more information on your rights, would like to exercise any right or have any queries relating to our processing of your personal data, or if you wish to make a complaint about how your data is being or has been processed, please contact:

The Data Protection Officer, Legal Services, The University of Birmingham, Edgbaston, Birmingham B15 2TT
Email: dataprotection@contacts.bham.ac.uk
Telephone: +44 (0)121 414 3916

You can also find out more from www.hra.nhs.uk/information-about-patients/ and by reading the information available here **www.hra.nhs.uk/patientdataandresearch**.

What will happen if I don't want to carry on with the study?

Your participation is completely voluntary. If you choose to take part but change your mind later, you are free to leave the study at any time, without giving a reason, and without your healthcare being affected. If you wish to withdraw from the study, please contact a member of the team (details are at the end of this document), However, please note that if you decide to withdraw more than 2 weeks after participating in an interview, any data already collected may still be used in the study.

What will happen to the results of the research study?

The information we collect will be analysed and the results will be presented in several ways:

- A short written summary of the results will be available on the OPTIMAL website hosted by the University of Birmingham, or can be sent out to participants should they prefer.
- A detailed report will be written and will be available for participants on the OPTIMAL website hosted by the University of Birmingham, or can be sent out to participants should they prefer.
- We will publish the results in academic journals.

Your details will not be shared at any time and you will not be identified in any of the results from the research.

Who is organising and funding the research?

The research study is funded by the National Institute for Health Research (NIHR). The research is sponsored and insured by the University of Birmingham.

Who has reviewed the study?

All research in the NHS is looked at by an independent group of people called a Research Ethics Committee (REC), to protect your interests. This study has been reviewed and given favourable opinion by South Central – Hampshire B Research Ethics Committee (REC

For peer review only

Reference: 22/SC/0210). Patients and public Involvement (PPI) members have been involved throughout all stages of the research study.

What happens next?

If you would like to participate in an interview, please contact a member of the research team whose details are given below.

The research team:
OPTIMAL Team
Institute of Allied Health Research,
University of Birmingham
B15 2TT

For more information, contact:
[name, email and contact number for study lead]

For peer review only

UNIVERSITY OF BIRMINGHAM

NIHR | National Institute for Health and Care Research

Supplementary Material 3

Study Title: **OPTIMIsing therapies, disease trajectories, and AI assisted clinical management for patients Living with complex multimorbidity (OPTIMAL study)**

Dear Colleague

We are writing to you on behalf of a team of researchers based at the University of Birmingham who are undertaking the above named study which aims to use artificial intelligence (AI) to produce computer programmes and tools that will help improve the treatment and choice of drugs in patients with clusters of multiple long-term conditions (complex multimorbidity or cMM).

As part of this study, the team are hoping to speak to healthcare professionals who care for patients with multiple (or more than four) long-term conditions (complex multimorbidity) to discuss their thoughts about how artificial intelligence (AI) may help professionals and patients manage these conditions. We will also be speaking to patients with long-term conditions to get their views.

We have attached a copy of the participant information sheet (PIS), to provide more details about the study. Before you decide whether you would like to take part in an interview, please read the enclosed participant information sheet that explains the study in more detail and what would happen if you agree to take part. If this is something you may be interested in taking part in, please email the address below.

Yours sincerely,

[name, email and contact number of study lead]

Institute of Applied Health Research
University of Birmingham
B15 2TT

For peer review only

Supplementary material 4

Case study for participants with multiple health conditions

Harinderjeet is 53 years old and owns a plumbing business with several employees. Harinderjeet has asthma, chronic lung disease, migraine, osteoarthritis (joint pain) and angina (chest pain). His work is very important to him as he feels responsible for his employees and wants to develop the business further. Most notably, his work helps to keep him distracted from this health conditions and pain.

He was diagnosed with asthma when he was 42 years, angina and osteoarthritis at age 45 years and then with lung disease two years later. As a result of his diagnoses he gave up smoking and has been smoke-free for the past five years. His GP told him he should exercise more and he tries to but the pain he experiences in his joints makes it difficult and he hardly has the time with running his business. When he last saw his GP last week he had his weight measured and his BMI was 29 kg/m^2 and his doctor said that he should think about losing some weight. Harinderjeet thinks that the worsening of some of his symptoms and health conditions are caused by side effects of some of the drugs he takes; he takes approximately 20 tablets a day.

Harinderjeet spends a large amount of time going to doctor and hospital appointments, both planned and unplanned contacts with the health care system about once a week. His is mainly in contact with his GP who will regularly review the drugs for his pain and asthma but then has separate appointments at the hospital for his lung disease and arthritis. He also sees a clinical nurse specialist for his angina. He started developing some severe pain in his tummy and told the doctor at the arthritis clinic who told him that it wasn't related to his arthritis and he should talk to his GP about it.

Q1. How does Harinderjeet's experience of being diagnosed with multiple health conditions compare with your experience? Explain some of the similarities and the differences.

Q2. How does Harinderjeet's experience of feeling that some of the worsening of his symptoms and being diagnosed with other health conditions are due to the medicines he is taking compare with your experience? Explain some of the similarities and the differences.

For peer review only

Case vignette for healthcare professionals - Janet

Janet is a white English woman aged 72 years who is a retired secretary living at home on her own.

From her records you can see:

She had an NSTEMI 3 years ago.

She has hypertension diagnosed over 10 years ago, type 2 diabetes diagnosed 6 years ago, and she has chronic kidney disease stage 3a.

BMI - 32.2 kg/m2.
Average home blood pressure is 134/82.
Renal function is stable with an eGFR of 55.
Diabetes is stable and reasonably well controlled with a Hba1c of 52.

She is a non-smoker and drinks occasionally. She does minimal exercise.

Her medications are:

Ramipril 10mg - ischaemic heart disease, CKD, diabetes, hypertension

Amlodipine -5mg hypertension

Glyceryl trinitrate spray - as required. - ischaemic heart disease.

Aspirin 75mg - ischaemic heart disease

Bisoprolol 10mg - ischaemic heart disease, hypertension

Atorvastatin 80 mg - ischaemic heart disease

Metformin - modified release 500mg BD - diabetes.

In today's consultation:

Her husband died 10 years ago, and she has a supportive family who live nearby but struggles with loneliness, especially at night time. She had bereavement counselling at the time of his death which helped her a lot. She used to go to church regularly until about 6 months ago and

For peer review only

has been a member of a choir for many years. However, over the past 6 months she has lost interest in going to these things any more (or joining the online versions even though she knows how). She has very poor sleep, reduced appetite, she can't concentrate on a book or TV programme. She feels quite "empty" and tired a lot of the time. She often thinks it might be better if she did not wake up the next day, but she does not have any plans for suicide. **You have diagnosed her with moderate to severe depression and she is now asking whether she could try some medication.** She knows her son has has been taking fluoxetine for many years for his depression and he found this really helpful. She is also willing to try some CBT.

Q1: What factors in the history (demographics, symptoms, past medical history, medications) would you consider when deciding how to manage her depression? (pt's age, sleep/appetite problems, other health problems, other medications, patient preference)

Q2: What factors or resources would you normally use to help make complex decisions like this? (own experience, NICE guidance, other guidance, patient preference?)

Q3: Assuming you decide to start an antidepressant, what would you recommend for Janet, and why?

Q4: The computer algorithm says Janet should take sertraline with a PPI, do you agree? (this is just an example, and I don't know if that would be the answer and whether it will give an explanation or not)

Q5: How would you feel about using the computer algorithm to make a decision like this compared with your usual practice? If you agree with the algorithm/ if you don't agree?

Very comfortable Mostly comfortable Not sure Mostly uncomfortable Very uncomfortable

Why?

Relevant NICE guidelines copied below:

From NICE guidelines; https://cks.nice.org.uk/topics/depression/management/new-or-initial-management/

For peer review only

Which antidepressants are recommended for people with chronic physical health problems?

- Prescribe a generic selective serotonin reuptake inhibitor (SSRI), such as sertraline or citalopram unless these are contraindicated or there is a significant drug interaction.
    - Do not use citalopram or escitalopram in people who are taking medication that could prolong the QT interval.
- For a brief summary on which antidepressants may be prescribed for people with chronic physical health problems, see Table 1. For more detailed information see appendix 16 of the full NICE guideline.

Table 1. Antidepressants recommended for people with a chronic physical health problem.

**Medication being taken for a chronic physical health problem**	**Antidepressants that should not normally be offered**	**Suitable options**
Nonsteroidal anti-inflammatory drugs (NSAIDs)	Selective serotonin reuptake inhibitor (SSRI) or a serotonin noradrenaline reuptake inhibitor (SNRI) (if no suitable alternative can be found, offer gastroprotection)	Mirtazapine, moclobemide, reboxetine, or trazodone
Aspirin	SSRIs and SNRIs (if no suitable alternative can be found, offer gastroprotection)	Mirtazapine.<br>When aspirin is used alone, consider trazadone or reboxetine.

For peer review only - http://bmjopen.bmj.com/site/about/guidelines.xhtml

Supplementary material 5

OPTIMIsing therapies, disease trajectories, and AI assisted clinical management for patients Living with complex multimorbidity (OPTIMAL study)

Interview guide: Patient Interview

This is the starting topic guide. The overarching objectives will remain the same, but questions and prompts will be developed as interviews are undertaken to incorporate any important themes emerging from the interviews.

Before the interview begins

- Ensure the participant has read the information leaflet
- Ensure the participant has had the opportunity to ask any questions about the research including issues about confidentiality, the findings of the research and where the research will be disseminated before being asked to agree to each item on the consent form.
- Start audio-recording
- Go through each item on the consent form and record their verbal consent. Explain that you will send/email a copy of the consent form for their records. They should already have a copy of the participant information sheet with details about the study, how to withdraw etc
- Explain that they don't have to answer all the questions just because they have consented to the interview, and that they can take a break or stop the interview at any time.
- Explain that you are there to understand more about their experiences and that they will have some time at the end of the interview to talk about any other issues that are important to them that may not have been covered by the questions.
- Check that they are happy to continue to be audio-recorded.
- Begin the interview.

TOPICS TO BE COVERED IN THE INTERVIEW

<u>Patient's reflections on living with/managing long-term conditions</u>

Could you tell me about your health conditions? Prompts – how long have you had conditions, how have they developed over time

What are your experiences of managing patients your long-term conditions? Prompts – medication, self-management, navigating primary/specialist care, relationships with clinicians/HCPs

Have you experienced any difficulties managing your conditions? Prompts -

- Side-effects from medication (impact on appetite, understanding which medications are for which condition; remembering to take medication)
- Multiple appointments for different conditions; opportunities to see GP/specialists; information sharing;

For peer review only

- Impact of conditions upon other activities e.g. work, time to socialise, leisure activities, access
- Impact upon relationships

Acceptability of AI in clinical decision making

Attitudes and understanding about AI in general terms (briefing guide of discussion points about AI)

Have you any thoughts about AI and how it is used in everyday activities?
Do you have any experience of AI being used to manage your healthcare?

Attitudes towards and perspectives about AI health care in managing long term conditions

In this study interview we want to explore the advantages and limitations of AI directed clinical decision making and compare this with doctors making decisions.

How do you feel about how clinical decisions are made about your healthcare? E.g. do you feel involved in decisions?

Do you think the use of AI (computer programs) in healthcare decision making could be beneficial? If so, how could it be used? If not, what are the reasons why?

Do you think that AI (computer programs) could improve clinical decision making?

Do you think that AI (computer programs) could help you manage your health conditions? If so, how may this benefit you and what would 'good' look like?

In which situations do you think AI (computer programs) should not be used?

What would your concerns be, if any?

How do you feel that using AI (computer programs) for clinical decision making may compare to clinician/patient making decisions about your health?

Can you think of ways AI (computer programs) making decisions about your care may be preferable to clinicians making decisions about your care?

Can you think of ways clinician/patient guided decisions about your care are preferable to AI directed decisions about your care? E.g. continuity of relationship; importance of relationship with HCP

What kind of questions might you want to ask before agreeing to treatment that was directed by AI?

What kind of things may influence your choice between AI or clinician/patient guided decisions about your care?

How confident would you feel if prescription decisions were directed by AI (computer program/predictive algorithm) rather than your doctor?
Very comfortable   Mostly comfortable Not sure   Mostly uncomfortable Very uncomfortable

For peer review only

Why?

Stage two: Vignettes – validity of AI in clinical decision making

We are developing a tool that will detect what other conditions a patient may develop in the future based upon their current health conditions. It will also provide information about what sort of medications should be prescribed (to achieve maximum health benefits and reduce side-effects etc)

Participants will have been sent a copy of the case vignette prior to the interview

We will present to the participant, a simulated patients disease clusters, to compare how aspects of the patient care management fit in with their own experiences:
The case vignette will present a simulated patients disease clusters, with 4 health conditions showing:

- How conditions developed
- What other conditions the patient developed
- Impacts of conditions upon their lifestyle and relationships etc
- How medications were managed
- How overall health management is navigated via the AI algorithm

How does the patient's experience of being diagnosed with multiple health conditions compare with your experience? Explain some of the similarities and the differences.

How does the patient's experience of feeling that some of the worsening of their symptoms and being diagnosed with other health conditions are due to the medicines he is taking compare with your experience? Explain some of the similarities and the differences.

What other things would you want to know about this patients to inform decisions about management of their treatment e.g lifestyle factors?

Would it be useful to know for example what conditions may develop in the future?

Would this impact upon things like lifestyle changes? E.g. dietary/exercise/self-monitoring regime

How would you feel about a computer programme predicting the medications you may benefit most from rather than your doctor?

Very comfortable Mostly comfortable Not sure Mostly uncomfortable Very uncomfortable

Does this case simulation reflect your own kind of experiences and preferences in terms of how your cMM are managed?

What do you think are the most important things that researchers need to consider when developing computer programmes to manage patient care?

Conclusion of interview

For peer review only

Now we've talked a bit about how AI could be used, how do you feel about it?

Thinking back to the difficulties you talked about in managing treatments, do you think AI could have an effect on this?

Thank you. That was my last question.

Is there anything you would like to add about the things we talked about but have not covered in the interview?

Any questions from interviewee

Reminder of study contact details and signposting

For peer review only

National Institute for Health Research

UNIVERSITY OF BIRMINGHAM

OPTIMIsing therapies, disease trajectories, and AI assisted clinical management for patients Living with complex multimorbidity (OPTIMAL):

Interview guide: Staff Interview

This is the starting topic guide. The overarching objectives will remain the same, but questions and prompts will be developed as interviews are undertaken to incorporate any important themes emerging from the interviews.

Before the interview begins

- Ensure the participant has read the information leaflet
- Ensure the participant has had the opportunity to ask any questions about the research including issues about confidentiality, the findings of the research and where the research will be disseminated before being asked to agree to each item on the consent form.
- Start audio-recording
- Go through each item on the consent form and record their verbal consent. Explain that you will send/email a copy of the consent form for their records. They should already have a copy of the participant information sheet with details about the study, how to withdraw etc
- Explain that they don't have to answer all the questions just because they have consented to the interview, and that they can take a break or stop the interview at any time.
- Explain that you are there to understand more about their experiences and that they will have some time at the end of the interview to talk about any other issues that are important to them that may not have been covered by the questions.
- Check that they are happy to continue to be audio-recorded.
- Begin the interview.

TOPICS TO BE COVERED IN THE INTERVIEW

<u>Managing patients with four or more long-term conditions</u>?

What are your experiences of managing patients with four or more long-term conditions? Prompt – what factors are important to consider when compared with patients with single diseases , comorbidties, other meds

For peer review only

What difficulties do patients with four or more long-term conditions have in managing their conditions and making decisions about treatment options?

Acceptability of AI in clinical decision making

Attitudes and understanding about AI in general terms (briefing guide of discussion points about AI) such as Amazon/ BBC iPlayer/ Spotify giving recommendations based on the existing data they have collected?

Do you have any experience of using AI in clinical practice?

Attitudes towards and perspectives about AI health care in managing cMM.

How do you think using AI directed clinical decision making may compare to usual practice?

Thinking about the impact on health care professionals ***and*** patients:

Thinking specifically about managing physical health conditions: Prompt? Diabetes, COPD, heart failure, CKD:

What are your thoughts on advantages and disadvantages of AI directed clinical decision making compared with your usual practice?

How do you think patients feel about AI directed clinical decision making?

What do you think patients may see as the advantages/ disadvantages of AI directed clinical decision making?

Are there examples where either using AI or using usual practice might be preferable?

What kind of things may influence your choice?

What are your thoughts on using AI directed clinical decision making with patients who have mental health conditions, e.g. depression/ anxiety?

What are your thoughts about using AI directed clinical decision making to make decisions about prescribing in physical health conditions (e.g., choosing the best medication for diabetes).

What are your thoughts about using AI directed clinical decision making to make decisions about prescribing treating mental health conditions (e.g., choosing the best antidepressant).

How confident would you feel if prescription decisions were directed by a predictive algorithm compared to your usual practice?

Very comfortable   Mostly comfortable Not sure   Mostly uncomfortable Very uncomfortable

For peer review only

Why?

Stage two: Vignettes – validity of AI in clinical decision making

We will present to the participant, a range of simulated patients from a range of disease clusters, to compare how aspects of the AI patient care management fit in with their own experiences a clinician:

What resources would you normally use to help make complex decisions like this? (NICE guidelines, own experience, other guidelines)

What medication changes would you recommend and why? (pt's age, sleep/appetite problems, other health problems, other medications, patient preference)

The computer algorithm says the patient should take X with the following explanation**, do you agree?**

How would you feel about using the computer algorithm to make a decision like this compared with your usual practice?

Very comfortable Mostly comfortable Not sure Mostly uncomfortable Very uncomfortable

Why?

How would you feel if the algorithm recommended a treatment you were not expecting? Would you over-ride if you disagree?

What would you do if a patient didn't want to follow the recommended treatment (i.e., would you look to over-rule it or stick by it?

Close of interview

Thank you. That was my last question. Is there anything you would like to add about the things we talked about but have not covered in the interview?

Supplementary materials 6

<u>Artificial Intelligence (AI) Background for participants</u>

Artificial Intelligence or AI is the term used to describe a computer system or algorithm that can conduct tasks that would normally require human intelligence.

There are examples of AI being used in our everyday lives, with applications and software such as Spotify, Amazon and BBC iPlayer. These 'apps' and on-line websites function by predicting what kind of music, TV programmes, or general purchases we may like or want. They do this by using a computer programme that can observe what categories and kinds of TV, music etc. we watched or bought in the past and use this data to predict what we are likely to choose in the future.

AI is also used in things like self-driving cars and language translations, and it is also being increasingly developed for use in healthcare. For example, AI has been developed that can screen the retina scans of patients with suspected diabetic eye disease. This is the leading cause of blindness in adults, and in many parts of the world, there are not enough doctors or health care professionals to undertake the work involved in diagnosing the condition. The AI trained system can diagnose the condition with the same accuracy as a trained healthcare professional.

In healthcare AI is also being developed to help us to predict how patients' health may progress in the future. It can also help to decide what are the best treatments and medications for the increasing number of people who are living with several long-term health conditions

Using the large amounts of anonymised patients' data available from GP and hospital electronic health records, AI can help us predict the life trajectories of people with multiple long-term conditions. For example, we know that people with diabetes may already have or are more likely to develop high blood pressure, heart disease and eye disease. These patients are usually prescribed medications to help manage all these conditions, but it can be difficult to ensure the best medications are prescribed for each individual patient based on the complexities of their own specific medical history, other characteristics and the variability in decisions made by different health professionals providing care.

Given the variability of factors that can influence health, having an efficient, accurate and easy to use AI programme that can take into account this variability and help 'predict' the best combination of medications, or what conditions may develop in the future may benefit patients and health care professionals.

For peer review only
